# The Effect of Cu^2+^ and Zn^2+^ Ions’ Nonbonded Interactions on the Aggregation of β‑Amyloid 1–16 and 25–35 Fragments—A Molecular Dynamics Simulation Study

**DOI:** 10.1021/acschemneuro.6c00057

**Published:** 2026-07-06

**Authors:** Rayla Kelly Magalhães Costa, Felipe Rodrigues Souza, Rudielson dos Santos Silva, Nicolás A. Rey, Andre Silva Pimentel

**Affiliations:** Department of Chemistry, 28099 Pontifical Catholic University of Rio de Janeiro, Rio de Janeiro, Rio de Janeiro 22453-900, Brazil

**Keywords:** β-amyloid aggregation, metal ions, molecular dynamics, free energy calculations, collective variables

## Abstract

Alzheimer’s disease is linked to the formation and accumulation of extracellular β-amyloid aggregates, with toxicity primarily attributed to soluble oligomeric species, as proposed by the oligomeric hypothesis. Concurrently, the metal ion hypothesis suggests that transition metal ions, such as Cu^2+^ and Zn^2+^, directly modulate the aggregation process and the structural stability of β-amyloid (Aβ) fragments. In this study, molecular dynamics simulations were employed to produce collective variables to investigate the effects of these ions and their concentrations on the aggregation of the β-amyloid 1–16 and 25–35 fragments in aqueous solution for the first time. The free energy profile of aggregation indicates that the presence of Cu^2+^ ions slightly decreases the energy associated with the aggregation of the β-amyloid 1–16 fragment, suggesting it acts as a modulator that partially stabilizes the oligomers. In contrast, we report for the first time that Zn^2+^ ions do not reduce the energy barrier for the aggregation of the β-amyloid 1–16 fragment in aqueous solution. Zn^2+^ displays a higher affinity for the acidic residues of Aβ_1–16_, establishing more frequent, yet less selective, contacts compared to those observed for Cu^2+^ ions. The main finding is that Cu^2+^ and Zn^2+^ ions modulate the early aggregation pathway of β-amyloid. In particular, at high concentrations, Cu^2+^ favors the formation of small, structurally ordered proto-oligomers enriched in antiparallel β-sheets, rather than simply increasing aggregate size, whereas Zn^2+^ does not exhibit an analogous effect under the conditions studied. This metal-induced stabilization of β-sheet-rich low-order oligomers, particularly pronounced for Cu^2+^, identifies early secondary structure transitions as the primary determinant of amyloid toxicity and a critical molecular event in Alzheimer’s disease. The evidence presented in this study enhances our understanding of the oligomeric and metal ion hypotheses of Alzheimer’s disease.

## Graphical abstract

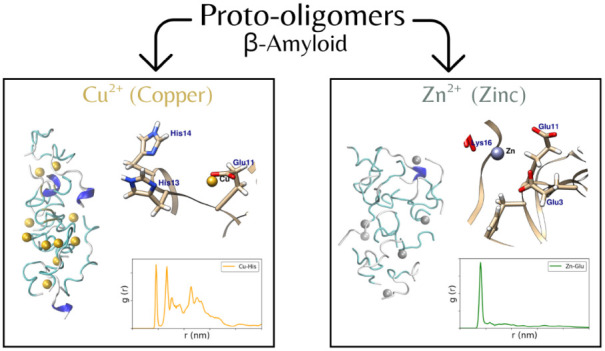

## Introduction

Alzheimer’s disease is a progressive neurodegenerative disorder, primarily affecting memory, cognitive functions, and behavior, eventually leading to loss of autonomy and incapacitation.^1,2^ It is characterized by brain atrophy in the hippocampus and cerebral cortex, neuropathological alterations such as the extracellular deposition of senile plaques of β-amyloid (Aβ) peptides, and intracellular neurofibrillary tangles composed of hyperphosphorylated tau protein.^3,4^ The Aβ peptides are generated from the cleavage of the amyloid precursor protein (APP) through the sequential action of the enzymes β-secretase in the endosomal pathway and γ-secretase in the plasma membrane.^4^ The peptides resulting from APP cleavage contain between 39 and 43 amino acid residues, with the most common variants being Aβ_40_ and Aβ_42_; the latter is more prone to aggregation and the formation of toxic deposits.^5–7^ These processes lead to synaptic dysfunction, neuronal death, and a progressive decline in brain functions. Currently, approved treatments for Alzheimer’s disease alleviate symptoms and slow its progression, but they do not cure the disease.^8^

Although several hypotheses have been proposed to explain the mechanisms of Alzheimer’s disease, this study focuses on two predominant hypotheses: the oligomeric hypothesis^6^ and the metal ion hypothesis.^9^ The oligomeric hypothesis, first proposed by Lambert et al. (1998), suggests that soluble oligomers of Aβ induce rapid synaptic impairment, an effect not seen with fibrils. Several studies since then have reinforced the idea that these soluble forms are highly toxic and are linked to the loss of synaptic plasticity, essential for the formation and consolidation of memory.,^3,10–12^

Recent findings highlight the role of endogenous metal ions in modulating Aβ oligomer toxicity, particularly redox-active metals like copper and iron, as well as nonredox-active metals such as zinc.^9,13–17^ Cu^2+^, Fe^3+^, and Zn^2+^ ions may enhance peptide aggregation and potentiate its neurotoxicity, suggesting that the interaction between Aβ and metals promotes peptide association by forming neurotoxic oligomeric species, thereby intensifying synaptic dysfunction.^14,17,18^ The fragment Aβ_1–16_ (by sequence, DAEFRHDSGYEVHHQK), which encompasses the N-terminal region of the peptide, contains histidine residues at positions 6, 13, and 14, which are essential for coordination with Cu^2+^ and Zn^2+^. The interaction with Cu^2+^ can generate reactive oxygen species, leading to oxidative stress even in the absence of fibrillar aggregation.^19–21^ Studying this fragment enhances our understanding of the structural changes induced by metal ions in the entire peptide. On the other hand, the Aβ_25–35_ fragment (by sequence, GSNKGAIIGLM), although small, maintains its ability to aggregate, form β-sheets, and induce neurotoxicity similar to that of the entire peptide.^22–24^ It aggregates quickly, is internalized by neurons, and causes cell death through mechanisms akin to those of Aβ_42_.^25–27^ Its compact structure makes it ideal for computational simulations of aggregation and the initial formation of oligomers, avoiding the complexity of the complete peptide.^28–30^

Among the computational approaches, molecular dynamics (MD) simulations enable the investigation of peptide behavior in solution and in the presence of metal ions like Cu^2+^ and Zn^2+^, which modulate the structure and toxicity of Aβ.^7,31,32^ To explore the relationship between conformational properties and aggregation-related intermolecular interactions, collective variables (CVs)^33^ are employed to facilitate the description of relevant system coordinates, allowing for efficient analysis of complex processes such as the nucleation and growth of oligomers, thereby enhancing our understanding of the essential molecular mechanisms of Aβ aggregation.^33,34^ This study aims to understand the effects of the presence of Cu^2+^ and Zn^2+^ ions on the aggregation of Aβ_1–16_ and Aβ_25–35_ fragments using MD simulations. The main goal is to elucidate how these interactions contribute to the molecular mechanisms associated with Alzheimer’s disease based on the oligomeric and metal ion hypotheses. It is important to emphasize that the treatment of transition metal ion interactions with the peptide fragments in classical molecular dynamics simulations is inherently approximate, as such approaches do not explicitly capture the partial covalency, variable coordination geometries, and electronic polarization effects that characterize metal coordination. In addition, it is also emphasized that the primary objective of the present study is not to model ion coordination chemistry at a quantum-mechanical level, but rather to investigate how nonbonding metal–peptide interactions modulate the aggregation behavior of Aβ fragments at relevant time and length scales. Within this framework, the use of a classical force field (where ions are represented as charged particles embedded in a water model) enables the systematic exploration of aggregation pathways and associated energetic profiles through the sampling of collective variables, which constitutes the central novelty of our work. We explicitly recognize that this approach provides a simplified description of metal–peptide interactions rather than a detailed account of coordination. We clearly state this limitation and restrict our conclusions to qualitative trends. Limitations of ion parametrization within classical force fields and their dependence on the solvent model have been discussed in the literature.^35^

## Methodology

MD simulations were used to investigate the aggregation process of Aβ fragments, specifically Aβ_1–16_ and Aβ_25–35_.^36,37^ The simulations were conducted with the software GROMACS 2024,^38^ employing the TIP4P water model.^39,40^ The initial conformations of Aβ_1–16_ and Aβ_25–35_ were selected from distinct experimentally resolved structures based on the availability of structurally validated models that best represented each fragment in an aggregation-relevant context. Specifically, the Aβ_25–35_ fragment was obtained from a preexisting structure optimized for this isolated sequence, ensuring consistency with prior studies that had characterized its intrinsic aggregation propensity. In contrast, the Aβ_1–16_ fragment was extracted from the full-length Aβ_42_ structure, preserving its native conformational context and maintaining the structural features associated with its role in metal binding and early aggregation events. We acknowledge that the use of different parent structures may introduce bias in the initial conformational ensembles, potentially influencing early-stage aggregation pathways and structural rearrangements. However, the primary objective of this study is not to perform direct structural comparisons between the two fragments, but rather to analyze trends within each system under varying ionic conditions. Therefore, our conclusions are drawn from relative differences observed within each fragment-specific simulation set and are interpreted with due consideration of the limitations associated with the initial structural selection.

The systems were composed of Aβ fragments at different concentrations, arranged in a cubic box with dimensions of 10 × 10 × 10 nm and solvated with approximately 32,000 TIP4P water molecules.^41^ A saline concentration of 0.15 mol L^–1^ was added by inserting Na^+^/Cl^–^ ion pairs, and additional counterions were included to neutralize the net charge of the system. Initially, an energy minimization step was performed using the steepest descent algorithm with a maximum of 50,000 steps or until a force of 100 kJ mol^–1^ nm^–1^ was achieved as a convergence criterion, considering van der Waals and electrostatic interactions with a cutoff radius of 1.2 nm and periodic boundary conditions.

To achieve the equilibration of the system, the canonical ensemble NVT (constant number of particles, volume, and temperature) was applied to adjust the temperature conditions without volume change, remove initial kinetic energy fluctuations, and ensure that the system temperature was around 310 K. The NVT simulations were performed with a simulation time of 5 ns, using the leapfrog algorithm as the integrator^42^ with a time step for integration of 2 fs, a temperature of 310 K, and controlled by the velocity rescaling thermostat (τ = 0.1 ps).^43^ The LINCS algorithm^44^ was used to constrain bonds between heavy atoms and hydrogens. Subsequently, the isothermal–isobaric ensemble NPT (constant number of particles, pressure, and temperature) was used to adjust the average pressure of the systems, reducing internal tensions and artificial pressure gradients. The simulation time, the integrator, the time step for integration, and the thermostat were the same as those used in the NVT step. The system pressure was set at 1 bar (with a time constant, τ = 2.0 ps) using exponential relaxation pressure coupling with the stochastic cell rescaling barostat (c-rescale).^45^

The production step was conducted for 500 ns under the same temperature and pressure conditions as those in the NPT step, using the same thermostat (τ = 0.1 ps)^43^ and barostat (τ = 2.0 ps).^45^ The long-range interactions and van der Waals interactions were treated by the Particle Mesh Ewald method^46^ with a real-space cutoff of 1.2 nm, a Fourier grid spacing of 0.16 nm, and a fourth-order interpolation scheme. Nonbonded interactions include a repulsion term, a dispersion term, and a Coulomb term. The repulsion and dispersion terms were described by the Lennard-Jones (6–12) potential, while electrostatic interactions between (partially) charged atoms were treated using the Coulomb potential.

The aggregation of Aβ peptide fragments was analyzed using a CV, utilizing the Colvar module^33,34^ of GROMACS (versions 2024 and later), which represented the degree of peptide aggregation, with a sampling frequency of 1000 steps, corresponding to a temporal resolution of 2 ps. A cutoff of 0.6 nm was applied, as recommended in the literature (typically 0.5–0.7 nm), to represent significant contacts between protein residues.^47–50^ The CV was defined based on the proximity between pairs of atoms belonging to two fragments. For its calculation, all pairs that enter the established cutoff are considered, with each pair counted upon crossing this limit. The contribution of each pair to the variable increases as its distance approaches the cutoff. Throughout the simulation, this variable reflects the proximity between the fragments, showing higher values when they are closer together and lower values when they are farther apart. This parameter facilitates monitoring the interaction between peptides and the formation of aggregates, aligning with the aim of this study of characterizing the initial mechanisms of oligomerization. This criterion is similar to the formalism of van der Waals interactions used in force fields, where a repulsive term prevents the overlap of atoms and an attractive term promotes physical proximity between them. Thus, the 0.6 nm cutoff serves as a realistic physical limit for intermolecular contact, enabling monitoring of relevant interactions between the peptides without including pairs that do not exhibit significant interaction.

For the analysis of the CV aggregation, a Python script was written to directly process the values obtained throughout the simulation. The data are organized into a histogram with 45 intervals (bins), from which the probability distribution P­(ξ) of the CV for each state of aggregation ξ is obtained. This distribution is normalized and used to calculate the free energy profile through the relation:

1 $$F\left(\xi \right){=-k}_{B}\mathrm{T ln P}\left(\xi \right)$$

where k_B_ is the Boltzmann constant and the temperature T = 310 K. The free energy F­(ξ) for each state of aggregation ξ is converted to kJ mol^–1^ for easier visualization. This procedure allows us to obtain the free energy profile, showing the most probable states of peptide interactions and the energetic costs related to the formation of each state of aggregation ξ (scripts available in the Supporting Information S1).

Radial distribution functions (RDF, g­(r)) were calculated to investigate the interactions between the metal ions (Cu^2+^ or Zn^2+^) and specific residues of the Aβ fragments. Using the RDF module implemented in GROMACS, the analyses were performed between the metal ions and the side-chain atoms of Asp1 and Asp7 (OD1/OD2), Glu3 and Glu11 (OE1/OE2), His6, His13, and His14 (ND1/NE2) residues, as well as the C-terminal carboxylate group of Lys16 (OC1/OC2). The g­(r) profiles were obtained from the last 50 ns of the MD trajectories, after ensuring structural equilibration, allowing us to quantify the relative probability of finding these residues at a distance r from the metal ions, in comparison with a homogeneous reference distribution.

## System Containing the Aβ_1–16_ Fragment

The structure of the Aβ_1–16_ fragment was obtained from the complete chain of Aβ_42_ available in the Protein Data Bank (PDB) (PDB ID: 2BEG).^37^ It was then prepared using PyMOL software.^51^ More specifically, the Aβ_1–42_ peptide was downloaded from the PDB file and processed to remove the Aβ_1–16_ fragment that came with several structures together and water that was also removed. Five conditions were evaluated using Aβ_1–16_ fragments: 10 fragments (0.0166 M) in the absence of metal ions; 10 fragments (0.0166 M) in the presence of 10 Cu^2+^ ions; 10 fragments (0.0166 M) in the presence of 20 Cu^2+^ ions; 10 fragments (0.0166 M) in the presence of 40 Cu^2+^ ions; 10 fragments (0.0166 M) in the presence of 10 Zn^2+^ ions; 20 fragments (0.0332 M) in the absence of metal ions; and 20 fragments (0.0332 M) in the presence of 20 Cu^2+^ ions. The OPLS-AA/M force field^52–54^ was used with compatible parameters for Cu^2+^ ions, allowing for an adequate representation of the interactions between this metal and the Aβ_1–16_ fragments. However, the parameter set available in OPLS-AA/M does not include parameters for all metal ions; therefore, for simulations containing Zn^2+^ ions, the AMBER14SB_OL24 force field^55,56^ was used. These choices were based on the availability of validated parameters for both Cu^2+^ and Zn^2+^ within the force field, ensuring greater reliability and a consistent, physically meaningful description of metal–peptide interactions. A Cu^2+^ system (10 Aβ_1–16_ fragments and 10 ions) was additionally simulated using AMBER14SB_OL24 ^55,56^ for direct comparison with the simulation containing 10 Zn^2+^ ions with 10 Aβ_1–16_ fragments in the same AMBER14SB_OL24 force field.^55,56^

## System Containing the Aβ_25–35_ Fragment

The structure of the Aβ_25–35_ fragment was obtained from the PDB (PDB ID: 1QYT)^36^ and later treated with the same procedure described for the Aβ_1–16_ fragment with the help of PyMOL software.^51^ For the Aβ_25–35_ fragment, three concentrations were evaluated: 10 fragments (corresponding to a peptide concentration of 0.0166 M), 20 fragments (0.0332 M), and 40 fragments (0.0664 M) in the absence of metal ions, along with a system containing 10 fragments (0.0166 M) in the presence of 10 Cu^2+^ ions. For the systems without ions, the OPLS-AA force field^53^ was used since this set provided widely validated parameters for amino acids and peptides, reliably reproducing conformational equilibria, intermolecular interactions, and structural behaviors relevant to aggregation processes. For the system containing Cu^2+^, the OPLS-AA/M force field^52,53^ was employed with compatible parameters for this ion.

The Aβ_1–16_ and Aβ_25–35_ fragments and metal ion concentrations used in this study are higher than physiological values. However, such conditions are widely used in molecular dynamics simulations to improve sampling and allow the observation of aggregation events within accessible simulation time scales. Since amyloid aggregation is a slow process at physiological concentrations, these events will not be observed within the typical duration of atomistic simulations.

## Results and Discussions

The following results describe the aggregation behavior of fragments Aβ_1–16_ and Aβ_25–35_ under different conditions. The analysis was conducted using the CVs obtained from MD simulations, allowing us to evaluate contact probability and associated free energies. Both the effects of the presence of Cu^2+^ and Zn^2+^ ions and the influence of the number of fragments on aggregation dynamics are presented, providing a combined analysis of metal interaction effects and fragment–fragment contact patterns that lead to the formation of oligomers.

The probability density distribution P­(ξ) (Figure 1) of the aggregation state (ξ) describes the relative frequency of the aggregated structural configurations sampled during the simulations as a function of the ξs. In all systems, the distributions extend over a continuous range of ξs, indicating that the aggregated ensembles explore a broad conformational space instead of a limited number of discrete configurations. The differences observed between the histogram bins reflect changes in the relative population of aggregated conformations induced by the system composition, while the global aggregated character is preserved in all cases; i.e., all bins accounted for in the histogram plot are aggregated and may be more or less compact. As observed below, the RDFs reveal frequent and geometrically diverse approaches of Cu^2+^ and Zn^2+^ toward charged residues and histidine. The absence of persistent coordination sites arises from the classical force-field description adopted, which emphasizes nonbonded interactions and, in the case of free ions in aqueous solution, does not explicitly represent the covalent character required for the stabilization of coordination complexes, as widely reported in experimental studies and quantum-based approaches.^57–60^ The broad peak associated with histidine means that Cu^2+^ and Zn^2+^ ions appear at various possible distances from the residue throughout the simulation. Thus, there is no single fixed contact geometry. The height of the peaks also changes between residues and between Cu^2+^ and Zn^2+^, which indicates that these contacts appear with different frequencies over time during the trajectory.

**Figure 1:**
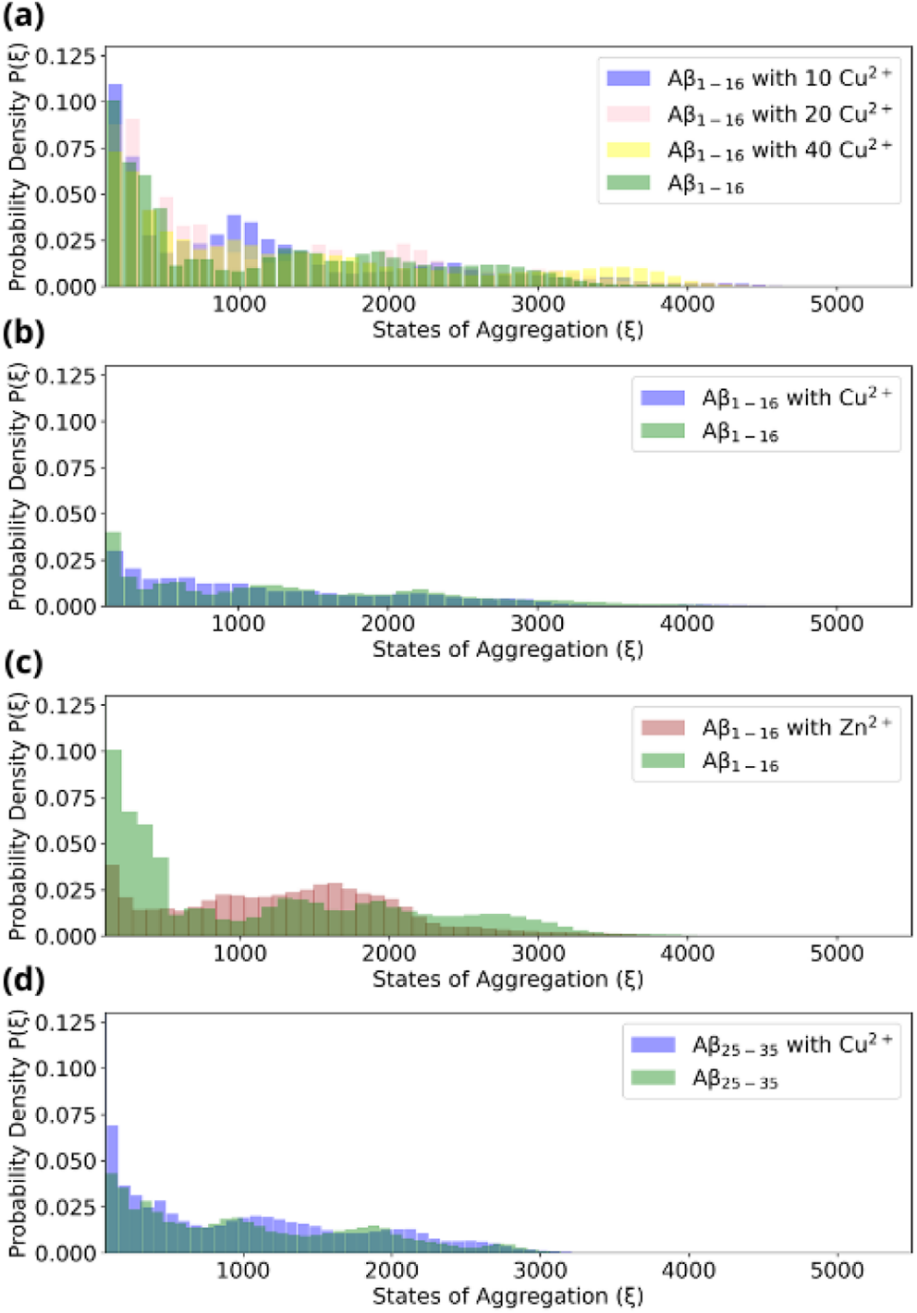
MD simulations were performed with β-amyloid fragments in aqueous solution to investigate the aggregation process under different conditions. The histogram shows the probability density distribution P­(ξ) of the aggregation states (ξ), calculated from the collective variable used to monitor peptide–peptide proximity. (a) Comparison between the system with 10 Aβ_1–16_ fragments in the presence of 10, 20, and 40 Cu^2+^ ions and the system without ions; (b) results for 20 Aβ_1–16_ fragments with and without 20 Cu^2+^ ions; (c) comparison between 10 Aβ_1–16_ fragments with 10 Zn^2+^ ions and without ions; and (d) analysis of 10 Aβ_25–35_ fragments with and without 10 Cu^2+^ ions. The first bar of each histogram was removed because it does not represent aggregated states.

For the system containing 10 Aβ_1–16_ fragments (Figure 1a), the presence of Cu^2+^ ions promotes a redistribution of the probability density P­(ξ). Although both systems explore a similar range of ξ values, differences in the relative weight of specific regions indicate that certain aggregated configurations are sampled with higher frequency in the presence of 10 Cu^2+^ ions. In the region of ξ between 3000 and 5000, associated with configurations less frequently accessed (i.e., rare events of aggregation), these states correspond to highly compact conformations that are thermodynamically disfavored but structurally informative regarding the upper limit of aggregate compaction. However, from a statistical-mechanical standpoint, such rare configurations must be interpreted with caution, as their low frequency implies limited sampling and potential sensitivity to initial conditions, precluding a robust estimation of their probability or mechanistic dominance. In particular, single or sparsely sampled occurrences cannot be assumed to represent reproducible features of the free energy landscape, nor can they support definitive kinetic or thermodynamic conclusions due to the lack of multiple barrier-crossing events. Accordingly, while these compact states indicate low population regimes, they are interpreted here as illustrative of the accessible structural limits of the system and the energetic cost associated with further compaction, rather than as representative or statistically converged configurations.

To further investigate the ions, the ion/peptide ratio was increased to 20 and 40 Cu^2+^ ions per 10 fragments. While the 10 Cu^2+^ ions with 10 fragments system may favor more compact aggregated states, higher concentrations (20 and 40 Cu^2+^ ions) may lead to the opposite behavior, with a clear reduction in the population of highly compact configurations in P­(ξ). This behavior is consistent with the concept of the electrostatic shielding effect. Following trends observed in recent coarse-grained studies, increased ion content shifts conformational ensembles toward more expanded states, less prone to association, as reported for intrinsically disordered proteins under varying salt conditions.^61^ These are less aggregated or less compact conformations with fewer contacts between them. In the literature,^61^ these conformations connect pathways spanning compact conformations to more expanded conformations, shifting chain conformations toward more expanded, less association-prone states. More generally, the effect of ion concentration on aggregation depends on the balance between competing nonbonded interactions, as also observed in other biomolecular systems.^62^ This interpretation is further supported by experimental studies showing that Cu^2+^ can modulate Aβ aggregation in a ratio-dependent manner, stabilizing less aggregated or nonfibrillar species at higher metal-to-peptide ratios.^63^

When the number of fragments is increased to 20 Aβ_1–16_ (Figure 1b), the probability distributions with and without Cu^2+^ become more similar along most of the ξ range. The distinct behavior observed at low and high concentrations (Figure 1a) reflects a change in the sensitivity of the aggregation collective variable rather than an inconsistency between the trajectories. At low concentrations, where intermolecular contacts are less numerous and more heterogeneous, ξ is more sensitive to metal-induced modulation of peptide interactions. In contrast, under high-density conditions, ξ becomes dominated by peptide–peptide contacts, and local ion-mediated effects are averaged out in this global descriptor. Although subtle differences in the relative population of specific regions can still be observed, the increase in system size results in a broader and smoother distribution, suggesting an increase in the conformational heterogeneity of the aggregated ensembles, which attenuates the effect of Cu^2+^ ions on the sampling of individual configurations.

A distinct redistribution of P­(ξ) is observed for the system containing 10 Aβ_1–16_ fragments in the presence of Zn^2+^ ions (Figure 1c). In comparison with the system without ions, the probability density shifts to different regions along ξ, indicating that Zn^2+^ ions may modify the relative frequency with which specific aggregated arrangements are sampled. The plot suggests that the Zn^2+^ ions may favor access to rare ξs in an almost negligible way when compared to the simulation without Zn^2+^ ions.

For the Aβ_25–35_ fragment (Figure 1d), the probability distributions obtained in the presence and absence of Cu^2+^ ions also differ in terms of the relative population along ξ, although the general shape and extension of the distributions remain similar. This behavior shows that the influence of Cu^2+^ ions has a limited role in this fragment. Methionine is the only residue with potential for direct interaction with Cu^2+^;^22,24^ however, the thioether sulfur acts as a weak ligand and does not support stable coordination in aqueous environments, where the strong hydration of the ion dominates. Within a classical force-field framework, Cu^2+^-methionine contacts are therefore expected to be transient and mainly governed by electrostatic proximity effects, leading to a modest influence on the aggregation behavior.

The free energy profiles F­(ξ) (Figure 2), derived from P­(ξ), describe the energetic cost associated with accessing different aggregated states along the collective variable for aggregation. In all analyzed systems, ξ values in the initial region (ξ < 1000) are associated with lower free energies, whereas higher ξ values (3000–5000) correspond to low-probability regions, reflected in a progressive increase in F­(ξ). These profiles indicate that aggregated states characterized by lower fragment–fragment proximity are thermodynamically more accessible, while highly compact configurations require a significant energetic penalty to be attained. However, it is important to note that the high-ξ region corresponds to rare events, and thus its quantitative characterization is inherently limited by the finite sampling of conventional molecular dynamics trajectories. In particular, the low population of these states implies that their associated free energy estimates may be sensitive to insufficient sampling, potential lack of ergodicity, and dependence on initial conditions, especially in the absence of multiple independent trajectories or enhanced sampling approaches. Consequently, these high-ξ regimes should be interpreted cautiously, not as statistically converged features of the free energy landscape, but rather as indicative of the upper energetic bounds associated with extreme compaction. Overall, these results demonstrate that metal ions primarily modulate the thermodynamic accessibility of compact oligomeric states rather than altering the fundamental aggregation pathway. Importantly, these profiles should be understood strictly as a description of relative energetic costs along the chosen collective variable and do not support direct mechanistic or kinetic inferences regarding aggregation pathways or temporal evolution.

**Figure 2:**
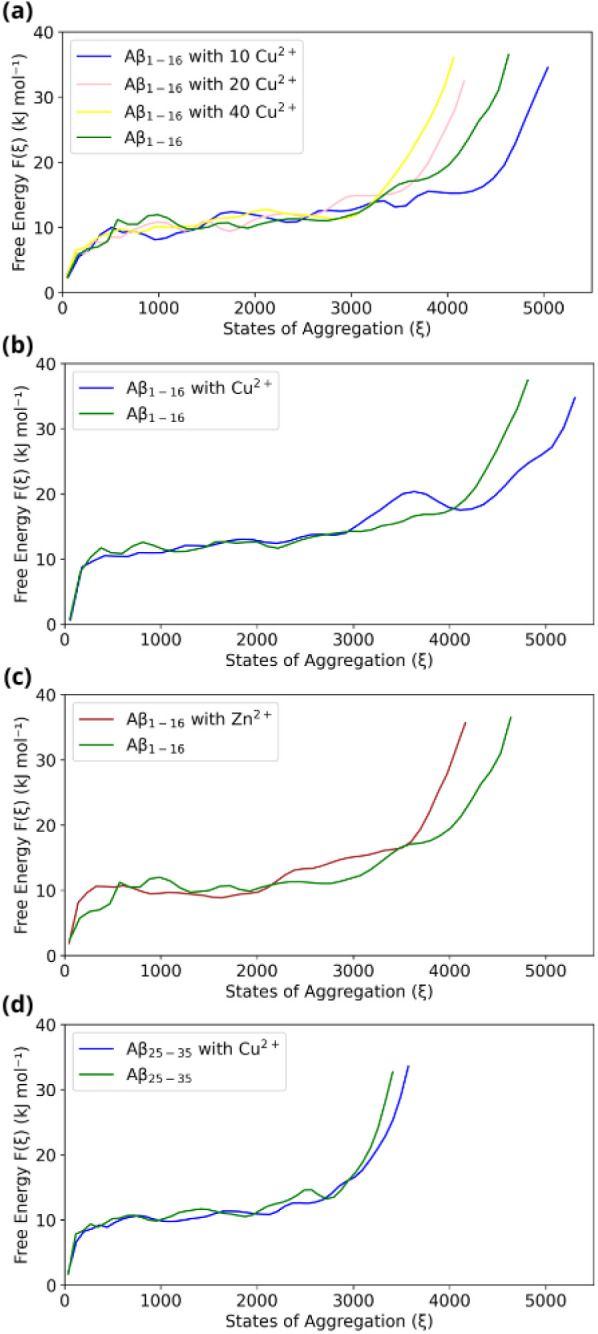
These profiles show the free energy (F = −k_B_T ln P­(ξ)) associated with accessing the different aggregation states in aqueous solution at 310 K, providing insight into the relative stability and energetic cost of each region along the collective variable ξ. (a) Comparison between the system with 10 Aβ_1–16_ fragments in the presence of 10, 20, and 40 Cu^2+^ ions and the corresponding ion-free system; (b) results for simulations with 20 Aβ_1–16_ fragments with and without 20 Cu^2+^ ions; (c) comparison of the free-energy landscapes for 10 Aβ_1–16_ fragments with 10 Zn^2+^ ions and without ions; and (d) analysis of the system containing 10 Aβ_25–35_ fragments with and without 10 Cu^2+^ ions.

For the system containing 10 Aβ_1–16_ fragments (Figure 2a), the comparison between the free energy profiles of aggregation in the presence and absence of Cu^2+^ ions reveals differences in the distribution of the lower energy states along ξ. In the presence of 10 Cu^2+^ ions, an alteration is observed in the ξ interval associated with the most energetically accessible states, indicating that Cu^2+^ ions may modulate the relative cost to access certain aggregated configurations. In the ξ region between 3000 and 5000, it is observed that the presence of Cu^2+^ ions may increase the number of lower energy states compared to the system without Cu^2+^ ions, indicating that they may modulate the thermodynamic accessibility of compact aggregated states. The low probability observed in the histogram suggests, however, that kinetic or entropic factors may limit the permanence of the system in these states (i.e., they are probably transient states). When increasing the ion/peptide ratio to 20 and 40 Cu^2+^ ions per fragment, it is observed that, in the same ξ region, the energetic cost associated with these configurations may become higher compared to the system with 10 Cu^2+^ ions, indicating a reduction in the accessibility of these compact states. This effect is more evident at higher ξ values, while at lower ξ the profiles remain similar. This behavior is consistent with the enhanced electrostatic shielding effect at higher Cu^2+^ ion/peptide ratios, which reduces effective peptide–peptide interactions. Experimental studies indicate that Cu^2+^ ions bind to Aβ predominantly in a 1:1 stoichiometry, forming a stable and well-defined complex under physiological conditions, with a conditional dissociation constant (K_d_) of approximately 0.035 μM.^64^ Cu^2+^ ions do not interact with Aβ only in a 1:1 stoichiometry, but can also form 1:2 and 2:1 complexes at higher concentrations, as described in the literature.^65^ Under physiological conditions, however, the predominant complex is 1:1.^65^

When the number of fragments is increased to 20 Aβ_1–16_ (Figure 2b), the free energy profiles become closer along most of the collective variable ξ, especially in the region of lower values (ξ between 0 and 3000). However, the system containing Cu^2+^ ions exhibits a delayed increase in energy compared to the system without Cu^2+^ ions, suggesting that they may stabilize the aggregate, at least partially. This result is in agreement with studies pointing to the role of Cu^2+^ ions as modulators of Aβ aggregation, acting not necessarily as direct promoters of fibril formation, but as influencers of the relative stability of oligomers.^66–68^ This behavior may be related to the ability of Cu^2+^ ions to coordinate specific residues, favoring slightly more compact or persistent conformations. This modulation can be relevant in the early stages of aggregation, although the more pronounced impact of Cu^2+^ ions is described for the full-length peptides, like Aβ_40_ ^69^ and Aβ_42._ ^70,71^

In the system containing Zn^2+^ ions (Figure 2c), a distinct modulation of the free energy profile is observed when compared to the system without Zn^2+^ ions. The presence of Zn^2+^ ions may alter the extent and slope of the F­(ξ) increase at higher ξ values (3000–5000), indicating differences in the energetic cost needed to access more compact ξs in the presence of Zn^2+^ ions. This shows that Zn^2+^ ions may not reduce the energetic barrier associated with certain rare ξs and act to decrease the thermodynamic accessibility to the rare states, which corroborates an MD study that indicated that the presence of Zn^2+^ ions in solution does not modify the aggregation rate of the peptide.^15^ The absence of significant variations in the observed aggregation for this system may indicate that the concentration of Zn^2+^ ions was not sufficient to promote amorphous precipitation, a process that requires an excess of metal ions,^72^ nor to shift the equilibrium in a way that substantially impacts the kinetics of oligomer formation. It is important to note that we do not modify the ion/peptide ratio for Zn^2+^, and this may constitute a determining parameter in defining both the pathway and the Zn^2+^/peptide aggregation rate, as suggested in the literature.^73,74^ Different from what was observed for the system with 10 Cu^2+^ ions, the presence of Zn^2+^ ions might not increase the frequency of occurrence of rare states and might not decrease the energetic cost needed to reach them when compared to the system without ions. This can be justified by visual analysis. In the system containing Zn^2+^ ions, it is observed that at most six ions participate directly in the formation of aggregates, while at certain moments, only two ions associate with the fragments, and the others remain in solution (Movie S3). In contrast, in the system with Cu^2+^ ions, all ions tend to interact with aggregates as soon as they are formed, with no observation at any moment of dispersed Cu^2+^ ions in solution (Movies S1, S2, S3, and S4). In contrast to Cu^2+^ ions, the interaction of Zn^2+^ ions with Aβ is more dynamic and cannot be described by a single well-defined binding affinity. Experimental studies show that Zn^2+^ ions initially yield a low-affinity complex (K_d_ ≈ 60 μM), which evolves over time into higher-affinity species (K_d_ ≈ 2 μM), associated with aggregation processes.^64^

To evaluate the possible influence of the force field on the observed behavior, an additional system containing Cu^2+^ ions was simulated using the same force field employed for Zn^2+^ ions. The comparison between the systems with Cu^2+^ ions (Movie S7) and Zn^2+^ ions (Movie S5) using the AMBER force field shows that the main features of the aggregation profiles are preserved. The probability distributions P­(ξ) indicate that Cu^2+^ ions yield higher-ξ aggregation states more frequently than Zn^2+^ ions (Figure S16a). Consistently, the free energy profiles F­(ξ) are similar up to ξ = 3000, while at higher ξ values (3500–5000) the system containing Zn^2+^ ions exhibits a steeper increase, indicating a higher energetic cost to access more compact states (Figure S16b). It is important to note, however, that this high-ξ region corresponds to rare-event regimes, and therefore its quantitative characterization is intrinsically limited by finite sampling. In particular, the low population of these configurations implies that differences in this regime may be sensitive to statistical noise, limited barrier-crossing events, and dependence on initial conditions, especially in the absence of multiple independent trajectories or enhanced sampling strategies. Accordingly, these results should not be overinterpreted in a strictly quantitative sense; rather, they provide qualitative evidence that Cu^2+^ ions facilitate access to more compact configurations relative to Zn^2+^ ions. Within this framework, the consistency of the observed trend across force fields supports the conclusion that the enhanced accessibility of compact states in the presence of Cu^2+^ ions is not an artifact of the force field choice, but instead reflects intrinsic differences in the interaction patterns of Cu^2+^ and Zn^2+^ ions with the peptide, while acknowledging the limitations associated with the description of rare events. The RDF profiles obtained for Cu^2+^ in the AMBER force field are similar indicating that the interaction patterns are preserved regardless of the parametrization (Figure S8).

For the Aβ_25–35_ fragments (Figure 2d), the free energy profiles of aggregation in the presence and absence of Cu^2+^ ions show similar behavior. A growing energy barrier is observed, which may be associated with the formation of more aggregated states, without relevant differences in the presence and absence of Cu^2+^ ions. The limited effect of Cu^2+^ ions on Aβ_25–35_ aggregation may likely reflect the reduced availability of canonical metal-binding residues in this fragment.

The literature shows that Cu^2+^ and Zn^2+^ ions have an affinity for specific residues in the fragment Aβ_1–16_, such as histidine (His, H), glutamate (Glu, E), and aspartate (Asp, D) (Figure S1), due to the presence of side-chain groups capable of interacting with these metal ions.^17,21,66,75,76^ In this context, while the CV captures global aggregation behavior, RDF analyses provide local structural insight and a quantitative description of the preferential proximity of these ions in relation to such residues, offering structural information about the metal ion–peptide interactions sampled along the simulations.

For the residues Asp1 and Asp7 (Figure 3d), well-defined peaks are observed at approximately 0.2 nm, indicating a higher relative probability of finding Cu^2+^ ions close to these residues. Similar behavior is observed for the residues Glu3 and Glu11 (Figure S2c), whose profiles show pronounced peaks at comparable distances, suggesting a preferential local organization of Cu^2+^ ions around these residues throughout the simulation. This result aligns with the high negative charge density of these residues, which favors short-range electrostatic interactions.^74,75^ In the case of histidine residues His6, His13, and His14 (Figure 3c), the RDF profiles show multiple peaks distributed over a wider range of distances, between 0.5 and 0.7 nm. This behavior indicates the sampling of different spatial arrangements between Cu^2+^ ions and these residues along the trajectory, reflecting the conformational diversity of the side chains and the local environment of the Cu^2+^ ions. This widened profile indicates that the interaction with some histidine amino acids is dynamic and flexible, suggesting that the Cu^2+^ ions access multiple modes of interaction instead of a single fixed site.^14,74,77–79^ Furthermore, the terminal carboxylate group of the Lys16 residue (Figure S2d) exhibits a well-defined peak around 0.25 nm, suggesting the possibility of electrostatic interactions, although it is less specific when compared to some histidine residues and in the absence of consolidated evidence for direct interaction reported in the literature so far. Figure 3 presents the representative structures derived from the simulation of a system containing 10 fragments and 10 Cu^2+^ ions. It illustrates the primary interaction modes between Cu^2+^ and Aβ_1–16_ residues and the corresponding radial distribution functions. Panels (a) and (b) display representative coordination environments, whereas panels (c) and (d) show RDFs describing interactions with His6, His13, and His14, and Asp1 and Asp7.

**Figure 3:**
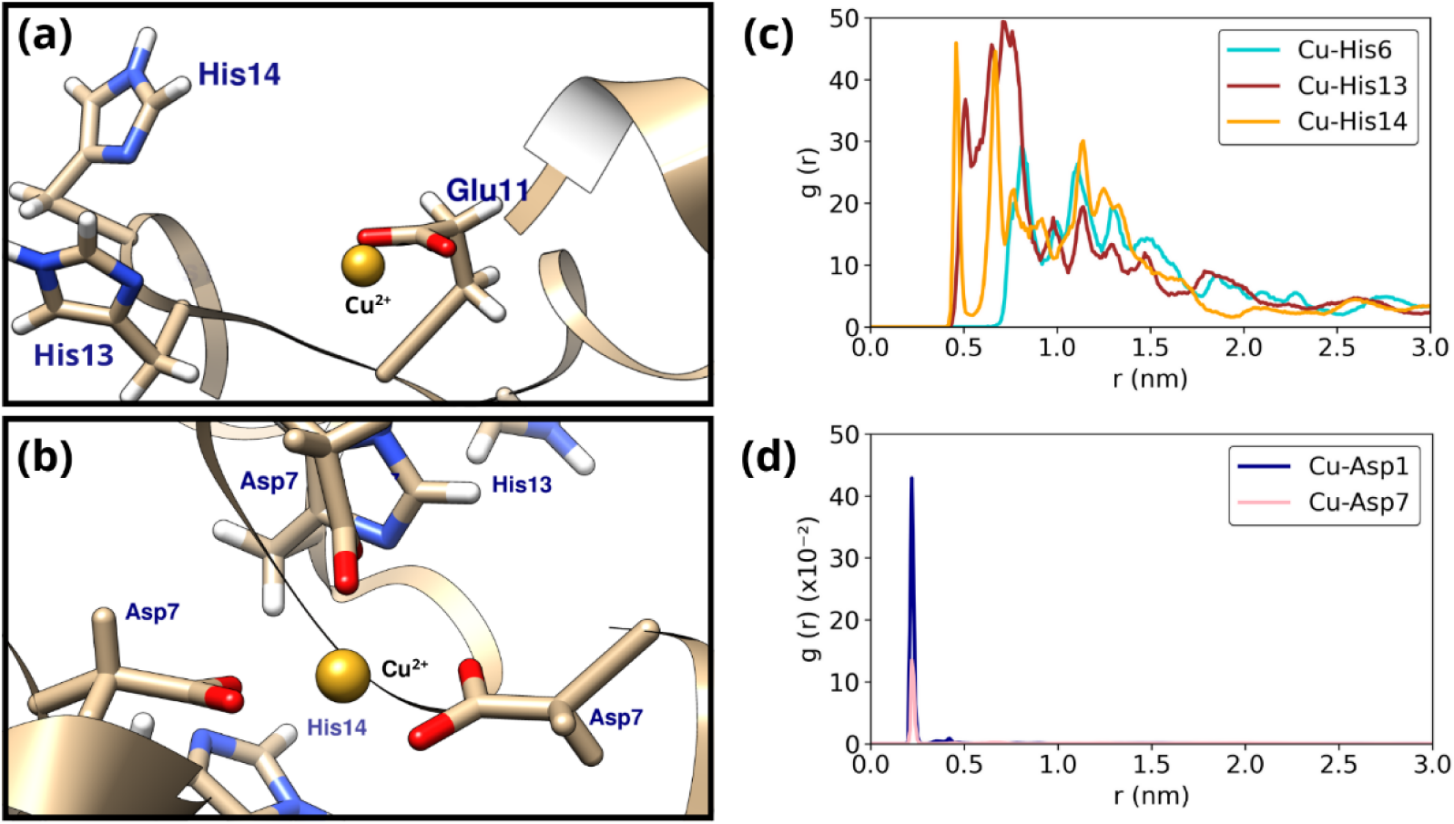
Representative structures obtained from simulations of the system containing 10 Aβ_1–16_ fragments and 10 Cu^2+^ ions, highlighting the main interaction modes observed between Cu^2+^ and peptide residues. (a) Interactions involving Glu11, His13, and His14; (b) interactions involving Asp7 and His14. The Cu^2+^ ion is represented as a golden sphere. (c–d) Radial distribution functions (RDFs) calculated from the final 50 ns of the simulation: (c) interactions of Cu^2+^ with histidine residues His6 (light blue), His13 (dark red), and His14 (orange); (d) interactions of Cu^2+^ with aspartate residues Asp1 (dark blue) and Asp7 (pink). The g­(r) values in panel (d) are multiplied by 10^–2^ for visualization purposes, whereas the values shown in panel (c) correspond to unscaled g­(r) values.

For the system containing 10 fragments of Aβ_1–16_ in the presence of 20 Cu^2+^ ions (Figures 4 and S3), the RDF profiles evidence distinct behaviors depending on the type of residue analyzed, reflecting the different modes of interaction of Cu^2+^ ions with the fragment. The histidine residues (Figure 4c) exhibit wider and more structured distributions, located in the range of approximately 0.5 to 2.0 nm. The presence of multiple peaks is observed, indicating the existence of different local arrangements accessed by the Cu^2+^ ions during the simulation. The interactions of Cu^2+^ ions with acidic residues, such as Asp and Glu (Figures S3c and 4d), are characterized by narrow and intense peaks at short distances, centered at approximately 0.2 nm, indicating direct contacts predominantly of an electrostatic nature. This behavior is consistent for Asp1, Asp7, and Glu11, suggesting a strong tendency for close interaction between the carboxylate groups and the Cu^2+^ ions. In a similar way, the Lys16 residue (Figure S3d) also presents a well-defined peak at about 0.2 nm, indicating the occurrence of contacts between the Cu^2+^ ions and the negatively charged terminal amino acid. Figure 4 displays representative structures obtained from simulations of a system containing 10 Aβ_1–16_ fragments and 20 Cu^2+^ ions and illustrates the main interaction modes observed between Cu^2+^ ions and peptide residues. Panels (a) and (b) show representative structures highlighting the main interaction environments involving acidic and histidine residues, whereas panels (c) and (d) present RDFs characterizing the interactions of Cu^2+^ ions with His6, His13, and His14, as well as with Glu3 and Glu11.

**Figure 4:**
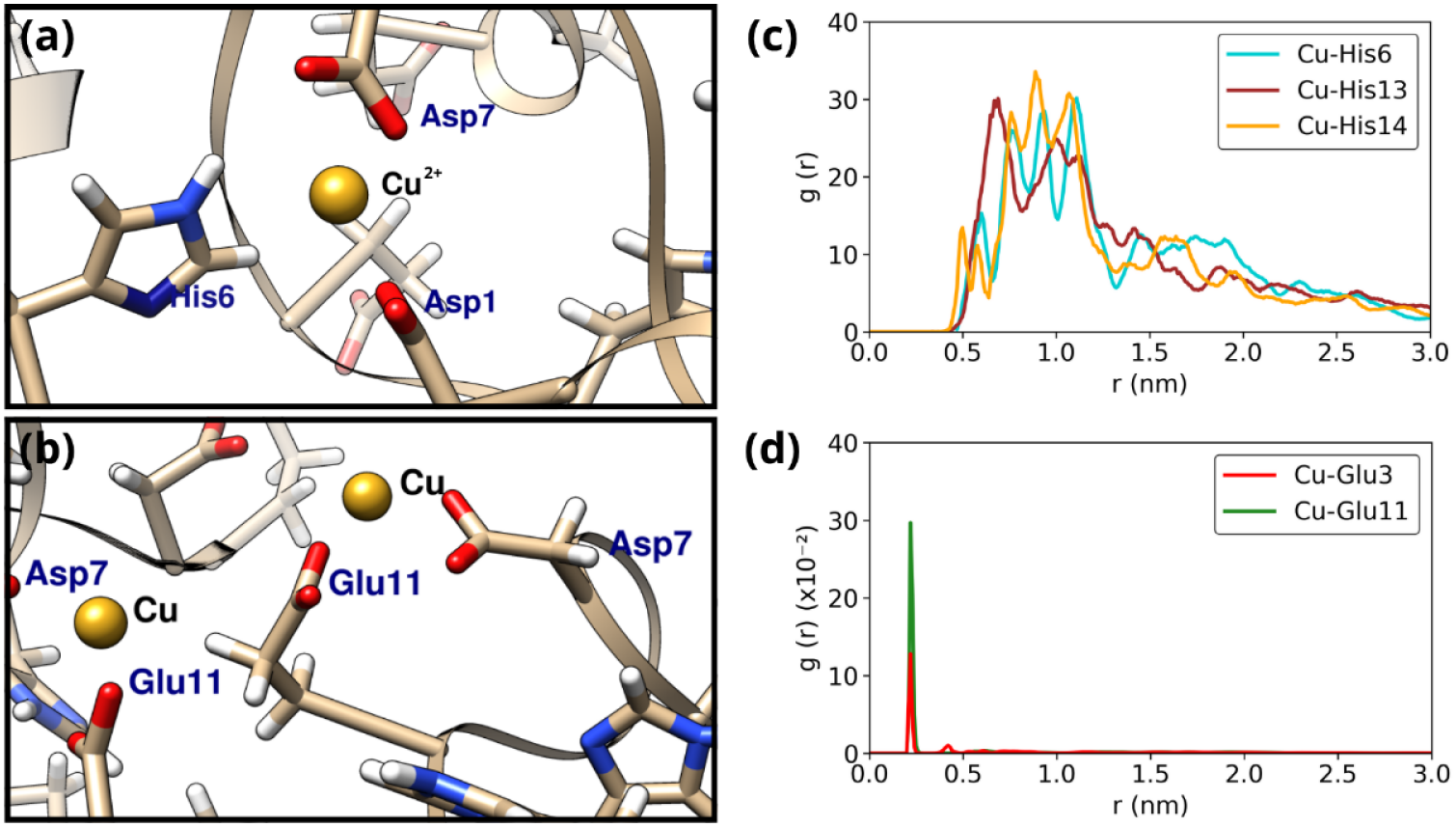
Representative structures obtained from simulations of the system containing 10 Aβ_1–16_ fragments and 20 Cu^2+^ ions, highlighting the main interaction modes observed between Cu^2+^ ions and peptide residues. (a) Interactions involving Asp1, Asp7, and His6; (b) interactions involving Asp7 and Glu11. The Cu^2+^ ion is represented as a golden sphere. (c–d) Radial distribution functions (RDFs) calculated from the final 50 ns of the simulation: (c) interactions of Cu^2+^ ions with histidine residues His6 (light blue), His13 (dark red), and His14 (orange); (d) the interaction of Cu^2+^ ions with Glutamate (Glu) at positions 11 (green) and 3 (red). The g­(r) values in panel (d) are multiplied by 10^–2^ for visualization purposes, whereas the values shown in panel (c) correspond to unscaled g­(r) values.

For the system containing 10 fragments of Aβ_1–16_ in the presence of 40 Cu^2+^ ions (Figures 5 and S4), the RDF profiles mantained general trends similar to the previous system, although differences were associated with the increased amount of Cu^2+^ ions in the medium. The histidine residues (Figure 5c) exhibit wide and structured distributions, located in the range of approximately 0.5 to 2.0 nm, reflecting a greater diversity of local arrangements and a more dynamic character of the Cu^2+^–imidazole interaction. The acidic residues (Asp1, Asp7, Glu3, and Glu11) (Figures 5d and S4c) present narrow and intense peaks at ∼0.2 nm, characteristic of direct Cu^2+^–carboxylate contacts, indicating predominantly electrostatic interactions and a more rigid character. This pattern is also observed for Lys16 (Figure S4d), whose interaction occurs via the terminal carboxylate group (C-terminal). Figure 5 displays representative structures obtained from simulations of a system containing 10 Aβ_1–16_ fragments and 40 Cu^2+^ ions and illustrates the main interaction modes observed between Cu^2+^ ions and peptide residues. Panels (a) and (b) show representative structures highlighting the main interaction environments involving acidic and histidine residues, whereas panels (c) and (d) present RDFs characterizing the interactions of Cu^2+^ ions with His6, His13, and His14, as well as with Asp1 and Asp7.

**Figure 5:**
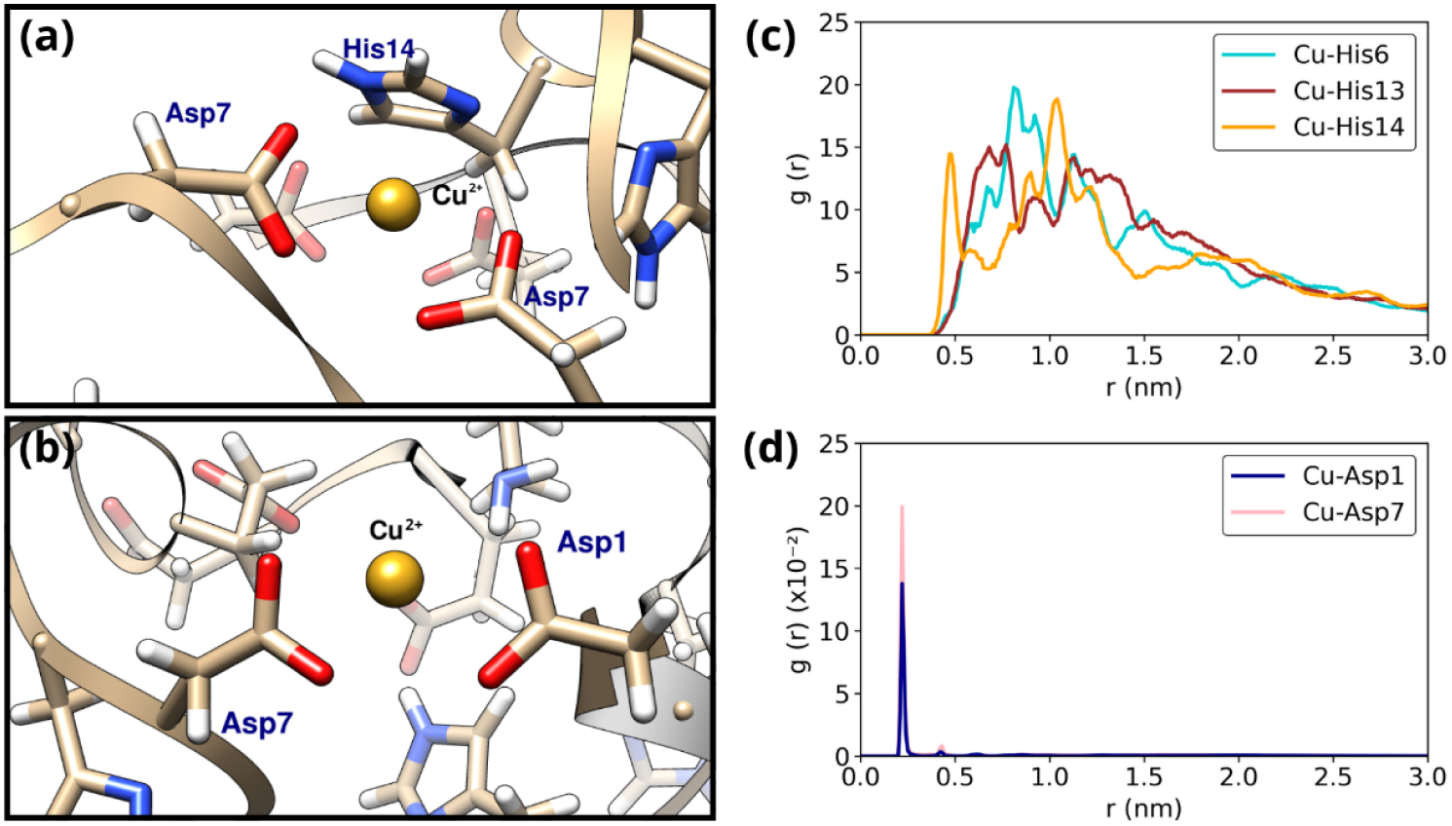
Representative structures obtained from simulations of the system containing 10 Aβ_1–16_ fragments and 40 Cu^2+^ ions, highlighting the main interaction modes observed between Cu^2+^ ions and peptide residues. (a) Interactions involving Asp7 and His14; (b) interactions involving Asp7 and Asp1. The Cu^2+^ ion is represented as a golden sphere. (c–d) Radial distribution functions (RDFs) calculated from the final 50 ns of the simulation: (c) interactions of Cu^2+^ ions with histidine residues His6 (light blue), His13 (dark red), and His14 (orange); (d) interactions of Cu^2+^ ions with aspartate residues Asp1 (dark blue) and Asp7 (pink). The g­(r) values in panel (d) are multiplied by 10^–2^ for visualization purposes, whereas the values shown in panel (c) correspond to unscaled g­(r) values.

For the system containing 20 Aβ_1–16_ fragments in the presence of 20 Cu^2+^ ions (Figures 6 and S5), the RDF profiles maintain general trends like those observed for the system with a smaller number of fragments, although with subtle differences associated with the increase in peptide concentration. The interactions of Cu^2+^ ions with Asp and Glu residues (Figures S5c and 6d) continue to be characterized by narrow peaks at short distances around 0.2 nm, indicating contacts dominated by electrostatic interactions. In contrast, the histidine residues (Figure 6c) show wider and more structured distributions, located in the range of approximately 0.4–2.0 nm, reflecting a larger variety of local arrangements accessed by Cu^2+^ ions, in agreement with the behavior observed in the 10-fragment system. The transient and flexible character of this interaction contrasts with the rigidity observed in the negatively charged residues, highlighting the dynamic nature of the interaction of Cu^2+^ ions with histidine in aqueous solution.^80,81^ For the terminal carboxylate group of the Lys16 residue (Figure S5d), a well-defined peak is again observed at 0.2 nm, suggesting occasional close contacts with the Cu^2+^ ions. Figure 6 displays representative structures obtained from simulations of a system containing 20 Aβ_1–16_ fragments and 20 Cu^2+^ ions and illustrates the main interaction modes observed between Cu^2+^ and peptide residues, together with the corresponding radial distribution functions. Panels (a) and (b) show representative structures highlighting the main interaction environments involving acidic and histidine residues, whereas panels (c) and (d) present RDFs characterizing the interactions of Cu^2+^ with His6, His13, and His14, as well as with Asp1 and Asp7.

**Figure 6:**
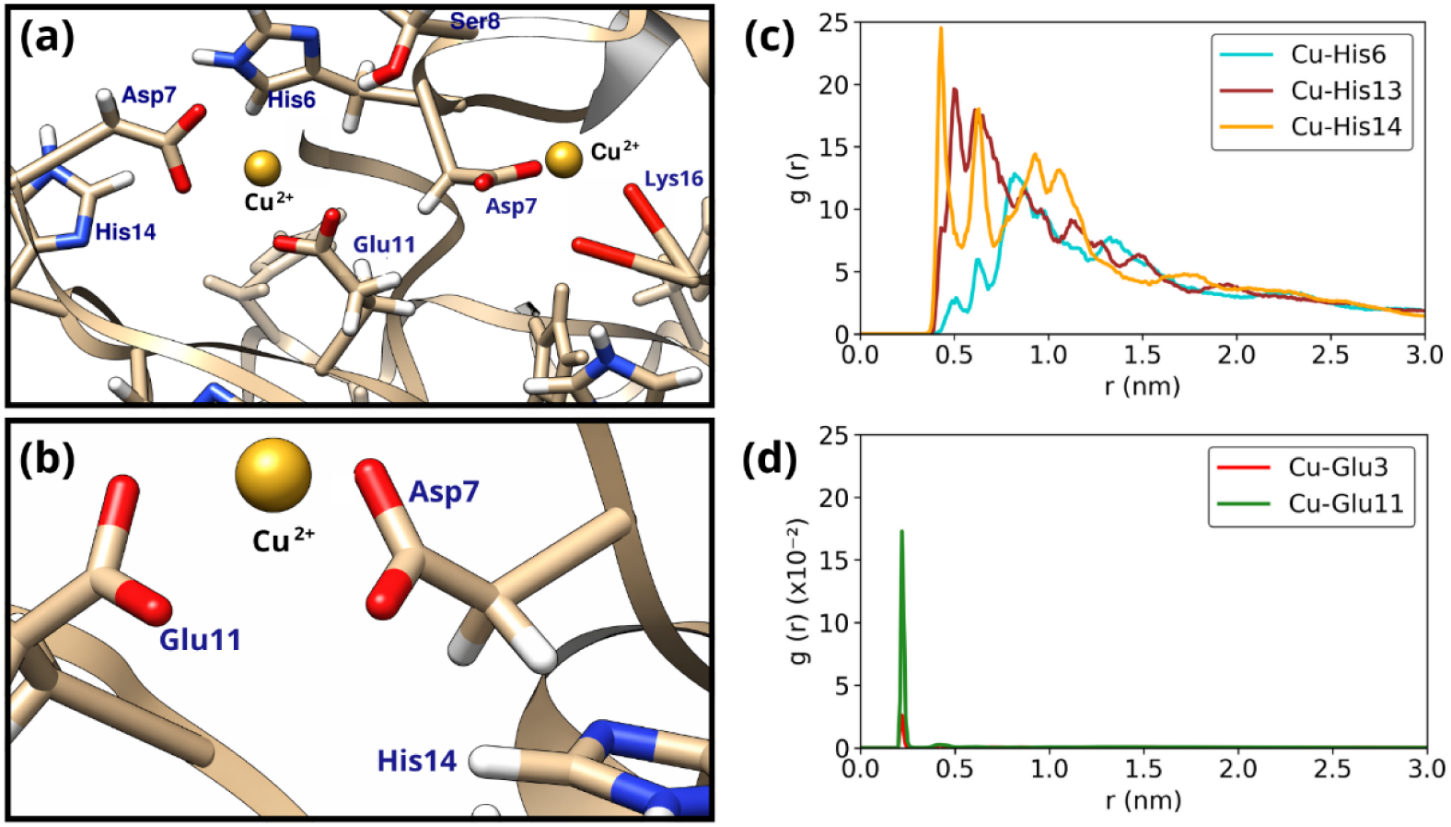
Representative structures obtained from simulations of the system containing 20 Aβ_1–16_ fragments and 20 Cu^2+^ ions, highlighting the main interaction modes observed between Cu^2+^ and peptide residues. (a) Interactions involving Asp7, Glu11, His6, His13, His14, and the terminal carboxylate of Lys16; (b) interactions involving Asp7, Glu11, and His14. The Cu^2+^ ion is represented as a golden sphere. (c–d) Radial distribution functions (RDFs) calculated from the final 50 ns of the simulation: (c) interactions of Cu^2+^ with histidine residues His6 (light blue), His13 (dark red), and His14 (orange); (d) interactions of Cu^2+^ with aspartate residues Glu3 (red) and Glu11 (green). The g­(r) values in panel (d) are multiplied by 10^–2^ for visualization purposes, whereas the values shown in panel (c) correspond to unscaled g­(r) values.

For the system containing 10 Aβ_1–16_ fragments in the presence of 10 Zn^2+^ ions (Figures 7 and S6), the RDF profiles reveal Zn^2+^ ion–residue proximity patterns that, although qualitatively comparable to those observed for Cu^2+^, show subtle differences in the spatial distribution of interactions. The residues Asp1 and Asp7 (Figure S6c) show peaks close to 0.4 nm, less intense than those observed for Cu^2+^, suggesting a weaker or more flexible interaction. In the residues His6, His13, and His14 (Figure 7c), the peaks are bifurcated at 0.3 and 2.0 nm, indicating multiple modes of interaction and higher conformational dispersion of Zn^2+^ ions. Glu3 and Glu11 (Figure 7d) present peaks of moderate intensity in comparison with Cu^2+^ ions at around 0.4 nm. The interaction pattern of Zn^2+^ ions suggests that they can explore more possible configurations in the peptide site but with slightly lower affinity, which is in accordance with their 3*d* ^10^ electronic configuration. Again, the terminal carboxylate group of the residue Lys16 (Figure S6d) also shows a notable peak around 0.25 nm, indicating the presence of additional electrostatic interactions between Zn^2+^ ions and the negatively charged terminal group. Figure 9 presents representative structures from simulations of a system containing 10 Aβ_1–16_ fragments and 10 Zn^2+^ ions, highlighting the main interaction modes between Zn^2+^ and peptide residues and the associated radial distribution functions. Representative structures in panels (a) and (b) emphasize interactions involving His13, His14, Glu3, Glu11, and the terminal carboxylate of Lys16, while panels (c) and (d) show RDFs describing interactions of Zn^2+^ with histidine and glutamate residues.

**Figure 7:**
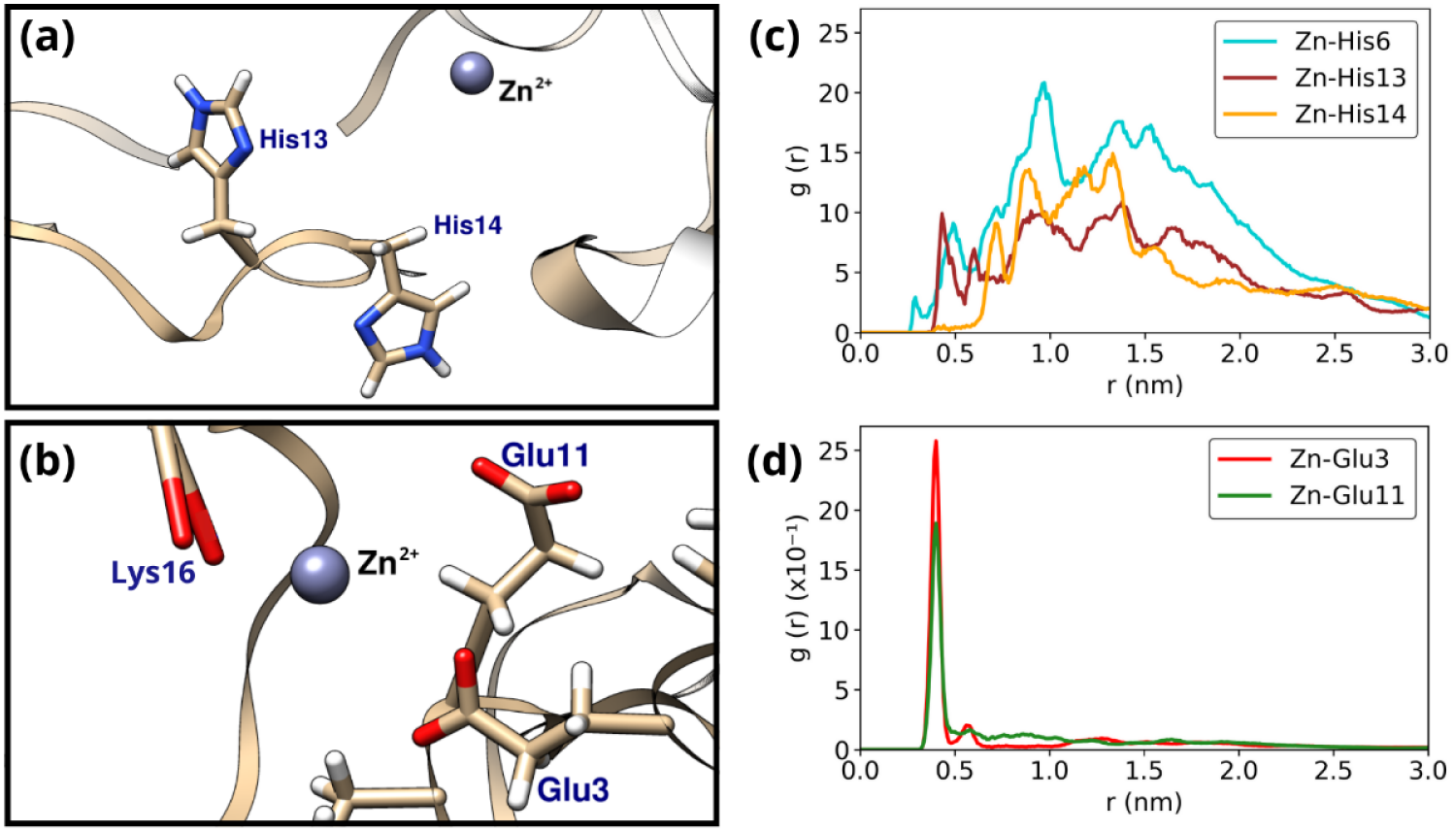
Representative structures obtained from simulations of the system containing 10 Aβ_1–16_ fragments and 10 Zn^2+^ ions, highlighting the main interaction modes observed between Zn^2+^ and peptide residues. (a) Interactions involving His13 and His14; (b) interactions involving Glu3, Glu11, and the terminal carboxylate of Lys16. The Zn^2+^ ion is shown as a blue sphere. (c–d) Radial distribution functions (RDFs) calculated from the final 50 ns of the simulation: (c) interactions of Zn^2+^ with histidine residues His6 (light blue), His13 (dark red), and His14 (orange); (d) interactions of Zn^2+^ with aspartate residues Glu3 (red) and Glu11 (green). The g­(r) values in panel (d) are multiplied by 10^–1^ for visualization purposes, whereas the values shown in panel (c) correspond to unscaled g­(r) values.

Overall, the comparison between Cu^2+^ and Zn^2+^ suggests that both ions access similar proximity regions with the analyzed residues, differing mainly in the relative distribution and in the geometric diversity of local contacts sampled along the simulation. This pattern shows that Zn^2+^ can favor the formation of aggregates by multivalent ionic bridges between different chains, but differs from the specificity typical of Cu^2+^ ions.^74^ This difference in selectivity can explain the distinct effects reported for Zn^2+^ and Cu^2+^ in the modulation of Aβ aggregation.^82–85^

For the system containing 10 Aβ_25–35_ fragments in the presence of 10 Cu^2+^ ions (Figure 8 and Figure S7), the profile associated with the Met35 residue (Figure 8b) shows a pronounced peak at short distances (0.5 nm), indicating that Cu^2+^ ions frequently access regions close to this residue during the trajectory. This behavior suggests recurrent contacts between the Cu^2+^ ions and the local environment of methionine, which may reflect transient approaches favored by the structural organization of the aggregate, without necessarily implying a specific or stable interaction. While the more diffuse profile observed for Asn27 (Figure S7b) indicates a less frequent and less localized proximity, these results reflect differences in the spatial accessibility of the residues within the aggregate and do not allow us to infer, by themselves, the formation of specific coordination interactions. Indeed, we have previously reported weak, axial Met–Cu^2+^ contacts when studying the interaction of this metal ion with the M112A mutant of the human PrP_103–112_ fragment.^86^ Still, the higher frequency of Cu^2+^ ions around Met35 suggests that this residue can contribute more significantly to Cu^2+^ ion–peptide contacts in the Aβ_25–35_ fragment, for which there is a lack of experimental and theoretical data in the literature.^66,87^ Figure 8 shows a representative structure obtained from simulations of a system containing 10 Aβ_25–35_ fragments and 10 Cu^2+^ ions, highlighting the main interaction modes between Cu^2+^ and residues of the fragment. Panel (a) illustrates the interaction of Cu^2+^ with the terminal carboxylate group of Met35, while panel (b) presents the corresponding RDF.

**Figure 8:**
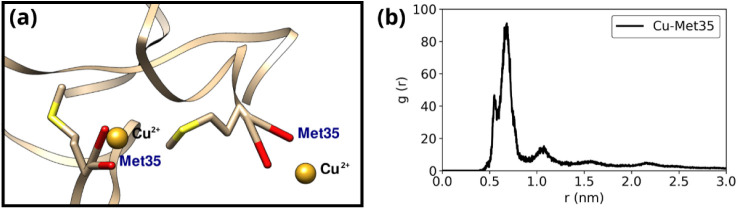
Representative structures obtained from the simulation of the system with 10 fragments and 10 Cu^2+^ ions, showing the main interaction modes observed between the Cu^2+^ ion and the residues of Aβ_25–35_. (a) Representative structure highlighting the interaction of the Cu^2+^ ion with the terminal carboxylate group of Met35 in the Aβ_25–35_ fragment; (b) radial distribution function (RDF) describing the interaction between the Cu^2+^ ion and the terminal carboxylate group of Met35.

In the present study, the simulated systems evolve toward proto-oligomeric assemblies, i.e., small and structurally dynamic metastable aggregates that represent transient intermediates in the early stages of amyloid self-assembly rather than fully equilibrated end-state oligomers.^88^ Within the accessible simulation time scale (500 ns), the systems do not reach global thermodynamic equilibrium, which is expected given the intrinsically slow kinetics and high free-energy barriers associated with oligomer association/dissociation processes. Instead, the trajectories sample a metastable ensemble characterized by continuous structural rearrangements and interconversion between larger aggregates (approximately 10 associated fragments) and smaller oligomeric clusters containing 3–4 fragments, indicating that the systems remain dynamically active rather than kinetically trapped in a single irreversibly aggregated state. Accordingly, the profiles reported as F­(ξ), obtained from the configurational probability distribution P­(ξ), should not be interpreted as rigorously converged equilibrium potentials of mean force or absolute free-energy surfaces in the enhanced-sampling sense, as would be required for quantitative characterization of fully reversible association pathways. Rather, these profiles provide an effective thermodynamic-like description of the relative configurational accessibility of aggregation states within the metastable ensemble sampled under identical simulation conditions. Therefore, the purpose of this analysis is comparative rather than absolute: to evaluate how Cu^2+^ and Zn^2+^ differentially modulate the accessibility of compact oligomeric configurations and reshape the relative population of aggregation states along the selected collective variable. No direct kinetic, mechanistic, or quantitatively converged thermodynamic conclusions are drawn from these profiles beyond this comparative framework. In this context, experimental studies indicate that interactions involving the N-terminal region of Aβ can influence interpeptide organization and favor structurally distinct oligomeric states. Although the present model is limited to noncovalent interactions and small Aβ fragments, the transient proto-oligomeric states observed here may reflect early characteristics of metal-influenced association.^89^

## Implications

The results discussed provide mechanistic insights into the early stages of Aβ aggregation, with particular emphasis on proto-oligomers’ formation modulated by Cu^2+^ and Zn^2+^ ions. By comparing systems with different peptide and metal concentrations, a nonlinear and highly concentration-dependent aggregation behavior emerges, challenging the intuitive assumption that higher concentrations simply promote larger aggregates. Instead, the data indicate that metal–peptide stoichiometry and local coordination environments critically reshape the aggregation landscape, influencing both proto-oligomer size distribution and secondary structure acquisition. At lower concentrations of Aβ_1–16_ and Cu^2+^, the aggregation process is dominated by the formation of large proto-oligomeric assemblies, typically comprising nine to ten peptides, often interacting with a comparable number of metal ions (representative aggregate structures are depicted in Figure 9a; further examples are available in Figure S9 and Movie S1). These assemblies appear to sequester nearly all available peptides into a single, highly populated proto-oligomeric state. Structurally, however, these species remain largely disordered, with peptides exhibiting intrinsic disorder or, at most, a modest propensity for α-helical conformations. Notably, there is an absence of β-sheet formation under these conditions, indicating that large oligomer size alone is insufficient to drive the conformational transition associated with amyloidogenicity.

**Figure 9:**
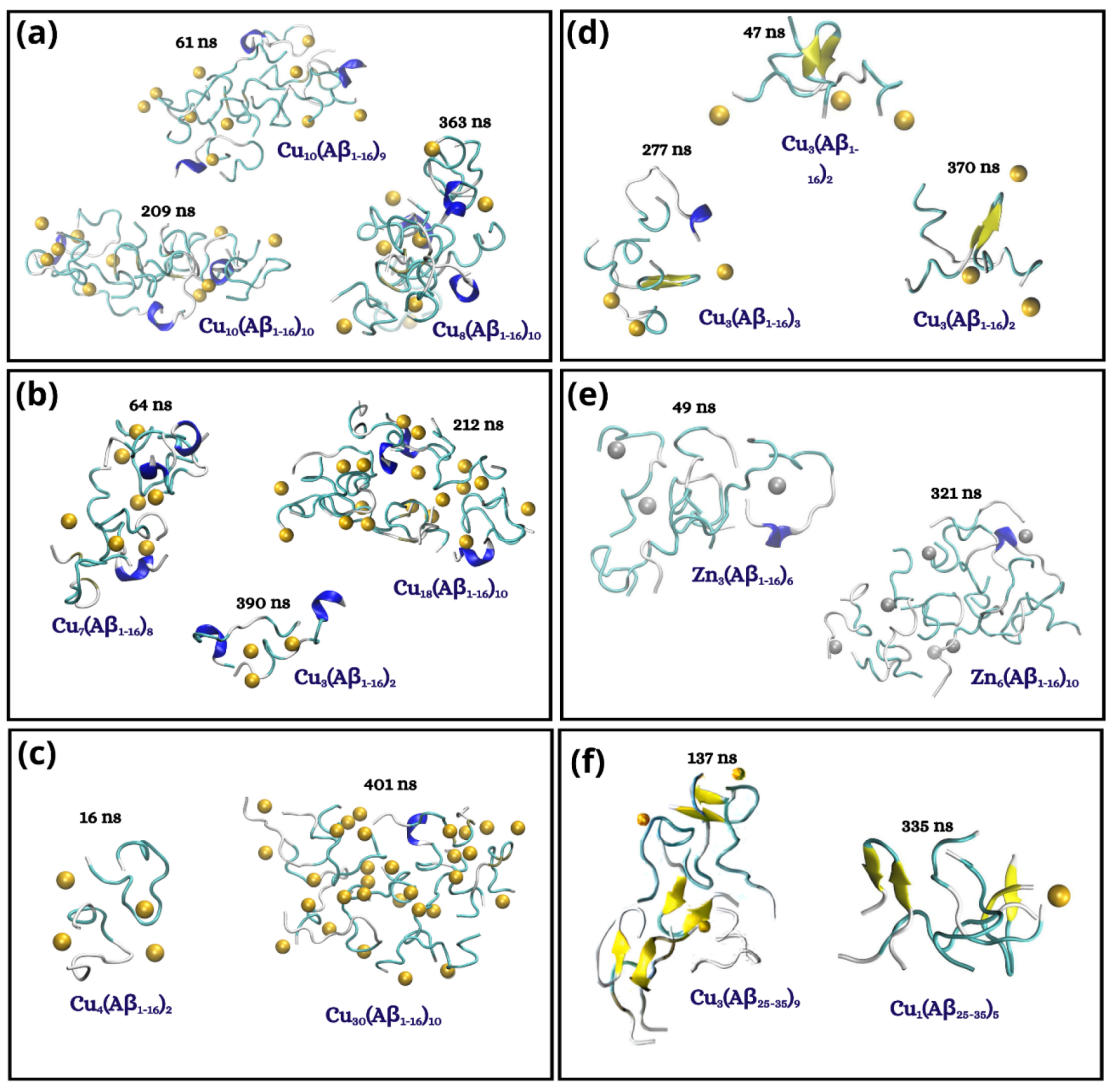
Snapshots from the MD trajectory, showing the formation of proto-oligomer species with varying aggregation numbers at different simulation times. (a) 10 Aβ_1–16_ fragments in the presence of 10 Cu^2+^ ions (gold spheres); (b) 10 Aβ_1–16_ fragments in the presence of 20 Cu^2+^ ions (gold spheres); (c) 10 Aβ_1–16_ fragments in the presence of 40 Cu^2+^ ions (gold spheres); (d) 20 Aβ_1–16_ fragments in the presence of 20 Cu^2+^ ions (gold spheres); (e) 10 Aβ_1–16_ fragments in the presence of 10 Zn^2+^ ions (blue sphere); and (f) 10 Aβ_25–35_ fragments in the presence of 10 Cu^2+^ ions (gold spheres).

In Figure 9b, the increase in the number of Cu^2+^ ions leads to a redistribution of proto-oligomer populations. Instead of a single dominant cluster as in the system with lower Cu^2+^ concentration, the system exhibits the coexistence of proto-oligomeric clusters of different sizes, although they still sequester most Cu^2+^ ions in solution (representative aggregate structures are shown in Figure 9b; other examples are available in Figure S10 and Movie S2). In Figure 9c, this behavior becomes more pronounced. Despite the formation of larger aggregates, the peptides remain largely disordered, with little consistent tendency toward the formation of α-helix structures, reinforcing that the increase in Cu^2+^ alone is not sufficient to induce structural organization under these conditions (representative aggregate structures are shown in Figure 9c; other examples are available in Figure S11 and Movie S3).

A striking qualitative shift occurs upon doubling both peptide and Cu^2+^ concentrations. Rather than stabilizing exclusively larger aggregates, the system frequently populates smaller proto-oligomeric species composed of two to four fragments, while larger assemblies involving a higher number of fragments are also observed (representative aggregate structures are depicted for Aβ_1–16_ in Figure 9d; further examples are available in Figure S12 and Movie S4). This redistribution toward lower-order proto-oligomers coincides with the robust emergence of Aβ_1–16_ condensation. Such condensation represents a critical molecular event, as it underpins the stabilization, aggregation competence, and neurotoxic potential of Aβ assemblies. These findings suggest that increasing metal and peptide concentrations promote conformational rearrangements that favor condensation within small oligomeric intermediates, rather than simple oligomer growth. From a mechanistic perspective, these observations support a model in which Cu^2+^ acts not merely as an aggregation agent but as a conformational catalyst, selectively stabilizing condensed Aβ_1–16_ arrangements within proto-oligomers for Aβ_1–16_. The preference for smaller, condensate Aβ_1–16_ assemblies under higher concentrations is particularly significant, as such species are widely implicated as the most neurotoxic forms of Aβ_1–16_. Thus, the toxic potential of Aβ_1–16_ aggregates appears to be more closely associated with their internal secondary structure and oligomeric order than with their overall size.

Comparative analysis with Zn^2+^ reveals both similarities and subtle, but important, differences. Like Cu^2+^, Zn^2+^ promotes, at low concentrations, the formation of large proto-oligomeric assemblies; however, these structures typically involve fewer interacting metal ions, suggesting weaker or less multivalent interactions (representative aggregate structures are depicted in Figure 9e; further examples are available in Figure S13 and Movie S5) This reduced metal involvement may modulate the stability and structural dynamics of Zn^2+^-induced oligomers, potentially leading to distinct aggregation pathways and toxic profiles.

Finally, studies involving the Aβ_25–35_ fragment further underscore the intrinsic amyloidogenic propensity of specific sequence regions. In this case, the Cu^2+^ ion interactions with the Aβ_25–35_ fragment are comparatively weaker than those with Aβ_1–16_ fragment. Yet, the peptide readily adopts antiparallel β-sheet conformations even in the absence of strong metal interactions (representative aggregate structures are depicted in Figure 9f; further examples are available in Figure S14 and Movie S6). This behavior highlights that while Cu^2+^ and Zn^2+^ ions can strongly modulate aggregation pathways, the primary sequence itself encodes a fundamental tendency toward β-sheet assembly, which ultimately defines the amyloid state.

Collectively, these findings emphasize that proto-oligomer formation, secondary structure acquisition, and Cu^2+^ and Zn^2+^ interactions are tightly coupled processes in Aβ aggregation. The promotion of small, β-sheet-rich proto-oligomers by Cu^2+^ and, to a lesser extent, Zn^2+^ provides a plausible molecular basis for metal-induced neurotoxicity in Alzheimer’s disease, positioning early metal-mediated conformational transitions as critical targets for therapeutic intervention.

For the metal ion-free systems containing 10 (0.0166 M), 20 (0.0332 M), and 40 (0.0664 M) Aβ_25–35_ fragments, investigated at different peptide concentrations (Figure S17), the P­(ξ) profiles may indicate that ξs with values smaller than ∼1000, associated with aggregated arrangements with lower relative proximity between fragments, are the most frequently sampled. As ξ increases, the probability continuously decreases, reflecting the higher energetic cost to access more compact, rare states. The free energy profiles may show a progressive increase of F­(ξ) at higher ξ values, with a gradual redistribution as the number of fragments increases, indicating that concentration may modulate the relative population of aggregated states. In all systems, rare ξs remain poorly accessed and energetically unfavored in the absence of Cu^2+^ and Zn^2+^ ions.

## Limitations

A fundamental limitation of the present study, as in many atomistic MD investigations of complex biomolecular systems, lies in the intrinsic difficulty of rigorously characterizing rare events from a limited number of trajectories. Rare events are, by definition, infrequently sampled and are often separated by substantial free-energy barriers. While such events are frequently of high mechanistic relevance, their statistical description within the framework of conventional MD is inherently constrained by finite sampling, particularly when relying on a single long trajectory.

From a statistical standpoint, the primary concern is the lack of representativeness associated with the sparsely observed configurations. Events that occur only once, or not at all, within a given trajectory cannot support robust estimation of their equilibrium probability nor can they be unambiguously distinguished from stochastic fluctuations.^90^ Consequently, any inference regarding their thermodynamic stability or mechanistic role remains inherently uncertain. This limitation is compounded by the pronounced sensitivity of rare events to initial conditions, including the starting conformations and velocity distributions. In practice, independent simulations initiated from different conditions may yield qualitatively distinct pathways or even temporal occurrences, thereby undermining reproducibility and limiting the generality of conclusions drawn from a single realization.^91^

A related issue concerns the incomplete exploration of the underlying free-energy landscape. Rare events typically correspond to transitions between metastable basins separated by high-energy barriers. In such cases, a single trajectory often remains confined to a restricted configurational space with an insufficient number of barrier-crossing events to ensure ergodic sampling. This precludes reliable estimation of free energy differences, transition rates, or state lifetimes and limits the ability to construct quantitatively accurate thermodynamic or kinetic models.^92^ Furthermore, the time-scale mismatch between standard MD simulations (typically spanning nanoseconds to microseconds) and the intrinsic time scales of rare-event processes (which may extend to microseconds, milliseconds, or beyond) introduces an additional layer of complexity. Observations of rare events within limited simulation windows may reflect either fortuitous sampling or transient fluctuations rather than representative dynamical pathways, thereby introducing potential time-scale bias.

An important practical consequence of these limitations is the risk of overinterpretation. Isolated, visually striking events may be tempting to interpret mechanistically. However, in the absence of recurrence or statistical validation, such observations cannot be considered dominant pathways but rather should be viewed as possible configurations within a broader ensemble. Accordingly, in the present work, rare events are not used as the basis for mechanistic conclusions. Instead, our analysis emphasizes statistically robust observables derived from equilibrated portions of the trajectories, including probability distributions and free-energy profiles along well-defined collective variables.

When discussed, rare configurations are explicitly treated as illustrative of the accessible structural extremes of the system, providing qualitative insight into the limits of conformational space and the associated energetic costs rather than serving as quantitatively converged features of the free energy landscape. Within this context, the conclusions drawn in this study are grounded in ensemble-like behavior, captured through time-averaged and distribution-based analyses rather than in single-transition narratives. Nevertheless, we acknowledge that a more rigorous characterization of rare event dynamics would require either multiple independent trajectories or the application of enhanced sampling methodologies.

## Conclusion

The results of this work are consistent with a substantial body of experimental evidence demonstrating the modulatory role of metal ions in the aggregation of Aβ_1–16_ and Aβ_25–35_ fragments. In this context, our findings provide a complementary molecular-level interpretation, indicating that Cu^2+^ and Zn^2+^ exert distinct effects on conformational stability and dynamic behavior that depend on the specific fragment and environment. In particular, RDF analyses reveal recurrent contacts of both Cu^2+^ and Zn^2+^ with negatively charged residues and histidine, highlighting differences in the relative frequency and conformations. Rather than introducing a new phenomenology, these results offer atomistic insights that help rationalize experimentally observed trends. However, no evidence of persistent coordination sites is observed, as the classical force-field description adopted favors nonbonded interactions and does not explicitly account for the covalent character required to stabilize metal–ligand coordination complexes. In this context, Cu^2+^ may display a more intricate modulatory role, influencing aggregation dynamics without necessarily promoting direct fibril formation, whereas Zn^2+^ may exhibit a predominantly indirect effect, remaining largely solvated and reducing the thermodynamic accessibility of rare, highly aggregated states. Despite the progress made, fully elucidating the interactions between Cu^2+^ and Zn^2+^ ions and the various oligomeric states of Aβ remains a challenge, and the observations presented here provide preliminary insights that may support future studies aimed at integrating experimental and theoretical approaches to further elucidate the role of metal ions in Aβ aggregation. Overall, the most significant finding is that Cu^2+^ and Zn^2+^ ions do not merely enhance Aβ aggregation in a quantitative manner but qualitatively redirect the early aggregation pathway. Enhanced Cu^2+^ and peptide concentrations favor the emergence of small proto-oligomers rather than large assemblies, while simultaneously inducing antiparallel β-sheet formation, a defining structural hallmark of neurotoxic amyloid species. Consistent with extensive experimental evidence demonstrating the pathogenic potential of small Aβ oligomers, our results do not directly address toxicity but instead provide structural insights into early aggregation events. In particular, the simulations suggest that metal ions, especially Cu^2+^, can modulate secondary structure within proto-oligomers, promoting condensed conformations independent of aggregate size. These observations offer a molecular-level perspective that may help rationalize experimentally observed relationships between metal binding, early structural organization, and toxicity. In future studies, the covalent component of these metal–peptide interactions will also be addressed in order to obtain a more comprehensive picture of the early stages of metal-mediated β-amyloid aggregation.

## Supplementary Material

















## Data Availability

Code, input data, and processing scripts for this paper are available on Zenodo (10.5281/zenodo.20032126). The data analysis scripts for this paper are also available in the format of an interactive notebook on Google Colab.

## Notes

Molecular dynamics trajectory of the system containing 10 Aβ_1–16_ fragments in the presence of 10 Cu^2+^ ions, illustrating the structural evolution and aggregation events throughout the simulation; the Cu^2^ ion is represented as a golden sphere (MPG)
Molecular dynamics trajectory of the system containing 10 Aβ_1–16_ fragments in the presence of 20 Cu^2+^ ions, illustrating the structural evolution and aggregation events throughout the simulation; the Cu^2+^ ion is represented as a golden sphere (MPG)
Molecular dynamics trajectory of the system containing 10 Aβ_1–16_ fragments in the presence of 40 Cu^2+^ ions, illustrating the structural evolution and aggregation events throughout the simulation; the Cu^2+^ ion is represented as a golden sphere (MPG)
Molecular dynamics trajectory of the system containing 20 Aβ_1–16_ fragments in the presence of 20 Cu^2+^ ions, illustrating the structural evolution and aggregation events throughout the simulation; the Cu^2+^ ion is represented as a golden sphere (MPG)
Molecular dynamics trajectory of the system containing 10 Aβ_1–16_ fragments in the presence of 10 Zn^2+^ ions, illustrating the structural evolution and aggregation events throughout the simulation; the Zn^2+^ ion is shown as a blue sphere (MPG)
Molecular dynamics trajectory of the system containing 10 Aβ_25–35_ fragments in the presence of 10 Cu^2+^ ions, illustrating the structural evolution and aggregation events throughout the simulation; the Cu^2+^ ion is represented as a golden sphere (MPG)
Molecular dynamics trajectory of the system containing 10 Aβ_1–16_ fragments in the presence of 10 Cu^2+^ ions, illustrating the structural evolution and aggregation events throughout the simulation, using the AMBER force field for comparison purposes; the Cu^2+^ ion is represented as a golden sphere (MPG)
The input data and analysis code; schematic representation of the Aβ_1–16_ peptide sequence and the main residues potentially involved in metal coordination; representative structures obtained from simulations of the system containing 10 Aβ_1–16_ fragments and 10 Cu^2+^ ions, highlighting the main interaction modes observed between Cu^2+^ and fragment residues and RDFs calculated from the final 50 ns of the simulation; representative structures obtained from simulations of the system containing 20 Aβ_1–16_ fragments and 20 Cu^2+^ ions, highlighting the main interaction modes observed between Cu^2+^ and fragment residues and RDFs calculated from the final 50 ns of the simulation; representative structures obtained from simulations of the system containing 10 Aβ_1–16_ fragments and 10 Zn^2+^ ions, highlighting the main interaction modes observed between Zn^2+^ and fragment residues and RDFs calculated from the final 50 ns of the simulation; representative structures obtained from the simulation of the system with 10 fragments and 10 Cu^2+^ ions, showing the main interaction modes observed between the Cu^2+^ ion and the residues of Aβ_25–35_ and RDF calculated from the final 50 ns of the simulation; snapshots from the molecular dynamics trajectory of 10 Aβ_1–16_ fragments in the presence of 10 Cu^2+^ ions; snapshots from the molecular dynamics trajectory of 20 Aβ_1–16_ fragments in the presence of 20 Cu^2+^ ions; snapshots from the molecular dynamics trajectory of 10 Aβ_1–16_ fragments in the presence of 10 Zn^2+^ ions; snapshots from the molecular dynamics trajectory of 10 Aβ_25–35_ fragments in the presence of 10 Cu^2+^ ions; probability distribution graph for the collective variable aggregation and energy required to access the aggregation states; comparison between simulations of aggregation of the Aβ_25–35_ fragment in aqueous solution with 10, 20, and 40 without ions (PDF)
