# Supplementary material for: The Effect of Cu2+ and Zn2+ Ions’ Nonbonded Interactions on the Aggregation of β‑Amyloid 1–16 and 25–35 Fragments—A Molecular Dynamics Simulation Study
Source: ACS Chem Neurosci. 2026 Jul 6;17(14):2603–19. doi: 10.1021/acschemneuro.6c00057 (PMC13377607; doi:10.1021/acschemneuro.6c00057)
Supplement: Supplementary file 8 [file cn6c00057_si_008.pdf]

The Effect of Cu^{2+} And Zn^{2+} Ions Non-Bonded Interactions on the Aggregation of β-Amyloid 1-16 and 25-35 Fragments – a Molecular Dynamics Simulation Study

Rayla Kelly Magalhães Costa, Felipe Rodrigues Souza, Rudielson dos Santos Silva, Nicolás A. Rey, and Andre Silva Pimentel.

Department of Chemistry, Pontifical Catholic University of Rio de Janeiro, Rio de Janeiro, RJ 22453-900 Brazil

*Corresponding author: a_pimentel@puc-rio.br

Supporting Information

S1: Colvars Module Configuration for the "Aggregation" Collective Variable.

```
colvarsTrajFrequency 1000
colvar {
  name aggregation_1_pep1&2
  #outputAppliedForce on
  coordNum {
    group1 { atomNumbersRange 1-155 }
    group2 { atomNumbersRange 156-310 }
    cutoff 0.6
    expNumer 6
    expDenom 12
  }
}
colvar {
  name aggregation_2_pep1&3
  #outputAppliedForce on
  coordNum {
    group1 { atomNumbersRange 1-155 }
    group2 { atomNumbersRange 311-465 }
    cutoff 0.6
```

```
        expNumer 6
        expDenom 12
    }
}
colvar {
    name aggregation_3_pep1&4
    #outputAppliedForce on
    coordNum {
        group1 { atomNumbersRange 1-155 }
        group2 { atomNumbersRange 466-620 }
        cutoff 0.6
        expNumer 6
        expDenom 12
    }
}
colvar {
    name aggregation_4_pep1&5
    #outputAppliedForce on
    coordNum {
        group1 { atomNumbersRange 1-155 }
        group2 { atomNumbersRange 621-775 }
        cutoff 0.6
        expNumer 6
        expDenom 12
    }
}
```

S2: Analysis Code for the Calculation of Free Energy from Collective Variables.

```
# Import necessary libraries.
import numpy as np
import matplotlib.pyplot as plt
from scipy.stats import gaussian_kde
from google.colab import files
# Constants
k_B = 1.380649e-23  # Boltzmann constant (J/K)
T = 310  # Temperature in Kelvin
kBT = k_B * T  # kB * T in Joules
# Function to convert Joules to kJ/mol for easier visualization
def joules_to_kj_per_mol(value):
    return value * 1e-3 * 6.022e23
cv_column_name = data.columns  # Change this to match your column name
cv_values = data[cv_column_name].values # Now access cv_values using the updated column list
# Convert all columns in cv_values to numeric, handling errors
for i in range(cv_values.shape[1]): # Iterate over columns
    try:
        cv_values[:, i] = pd.to_numeric(cv_values[:, i], errors='raise').astype(float)
    except ValueError:
        # Replace invalid values with NaN
        cv_values[:, i] = pd.to_numeric(cv_values[:, i], errors='coerce')
        # If you prefer to drop rows with invalid values, you can use:
        # data = data.dropna(subset=[cv_column_name[i]]) # Drop rows with NaN in this column
# Build Histogram
num_bins = 45  # Number of bins for the histogram
hist, bin_edges = np.histogram(cv_values, bins=num_bins, density=False)
bin_centers = 0.5 * (bin_edges[:-1] + bin_edges[1:])
```

```
# Normalize histogram to get P(ξ)
P_xi = hist / np.sum(hist)
# Step 3: Calculate Energy
# Avoid log(0) by replacing zeros with a very small value
P_xi[P_xi == 0] = 1e-10
F_xi = -kBT * np.log(P_xi)
# Convert F(ξ) to kJ/mol for better visualization
F_xi_kj_mol = joules_to_kj_per_mol(F_xi)
```

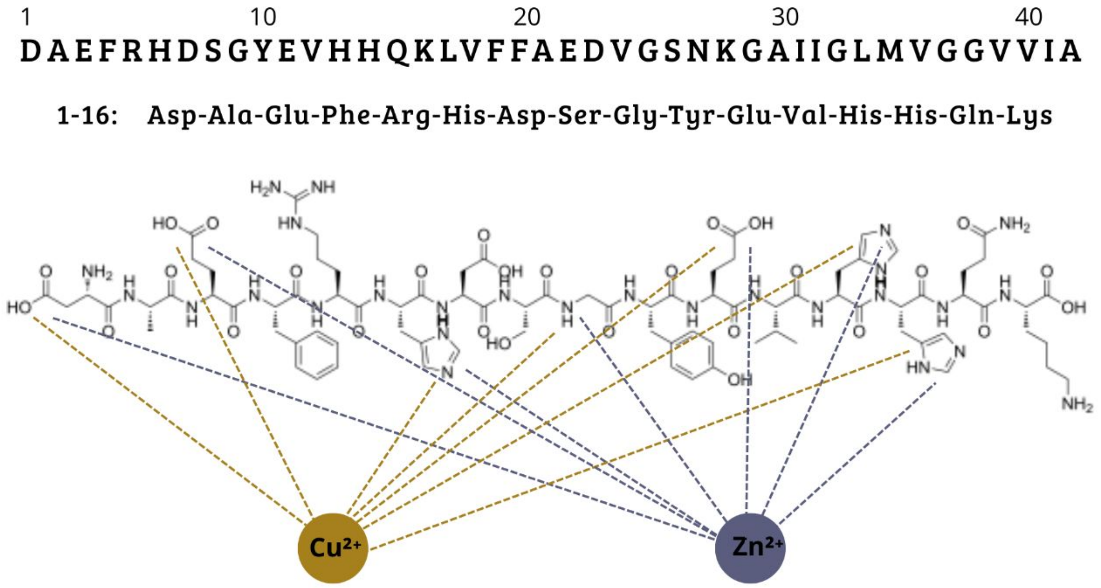

Figure S1: Schematic representation of the Aβ_{1-16} peptide sequence and the main residues potentially involved in metal coordination. The dashed lines show typical interactions between Cu^{2+} (golden) and Zn^{2+} (blue) ions with His, Glu, and Asp residues. These regions act as preferred coordination sites and may influence the structure and aggregation behavior of the β-amyloid peptide.

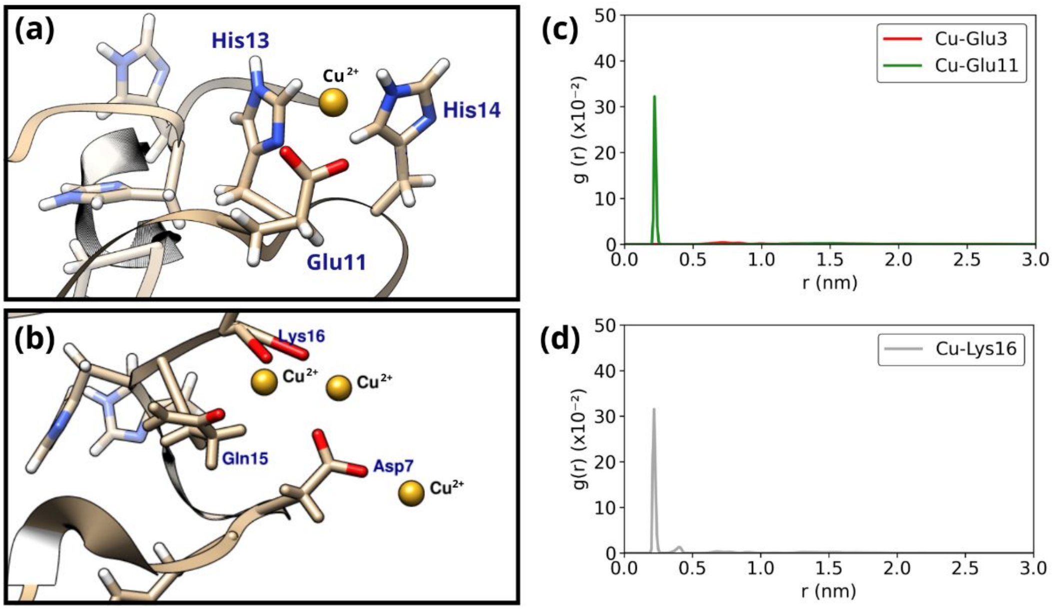

Figure S2. Representative structures obtained from simulations of the system containing 10 $A\beta_{1-16}$ fragments and 10 Cu^{2+} ions, highlighting the main interaction modes observed between Cu^{2+} and fragments residues. (a) Interactions involving Glu11, His13 and His14; (b) interactions involving Asp7 and the terminal carboxylate of Lys16. The Cu^{2+} ion is represented as a golden sphere. (c-d) Radial distribution functions (RDFs) calculated from the final 50 ns of the simulation: (c) the interaction of Cu^{2+} with Glutamate (Glu) at positions 11 (green) and 3 (red); (d) the interaction of Cu^{2+} with the terminal carboxylate of Lysine (Lys) at position 16 (gray). The g(r) values in panel (c) and (d) were multiplied by 10^{-2} for visualization purposes.

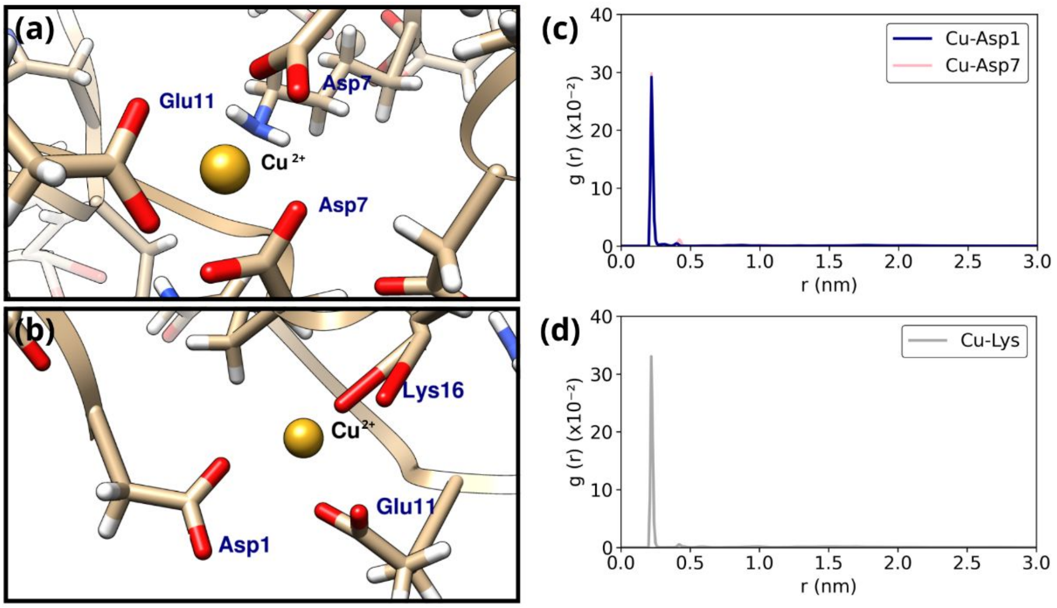

Figure S3. Representative structures obtained from simulations of the system containing 10 $A\beta_{1-16}$ fragments and 20 Cu^{2+} ions, highlighting the main interaction modes observed between Cu^{2+} and fragments residues. (a) Interactions involving Asp7 and Glu11; (b) interactions involving Asp1, Glu11 and the terminal carboxylate of Lys16. The Cu^{2+} ion is represented as a golden sphere. (c-d) Radial distribution functions (RDFs) calculated from the final 50 ns of the simulation: (c) the interaction of Cu2+ with Aspartate (Asp) at positions 7 (pink) and 1 (dark blue); (d) the interaction of Cu^{2+} with the terminal carboxylate of Lysine (Lys) at position 16 (gray). The g(r) values in panel (c) and (d) were multiplied by 10^{-2} for visualization purposes.

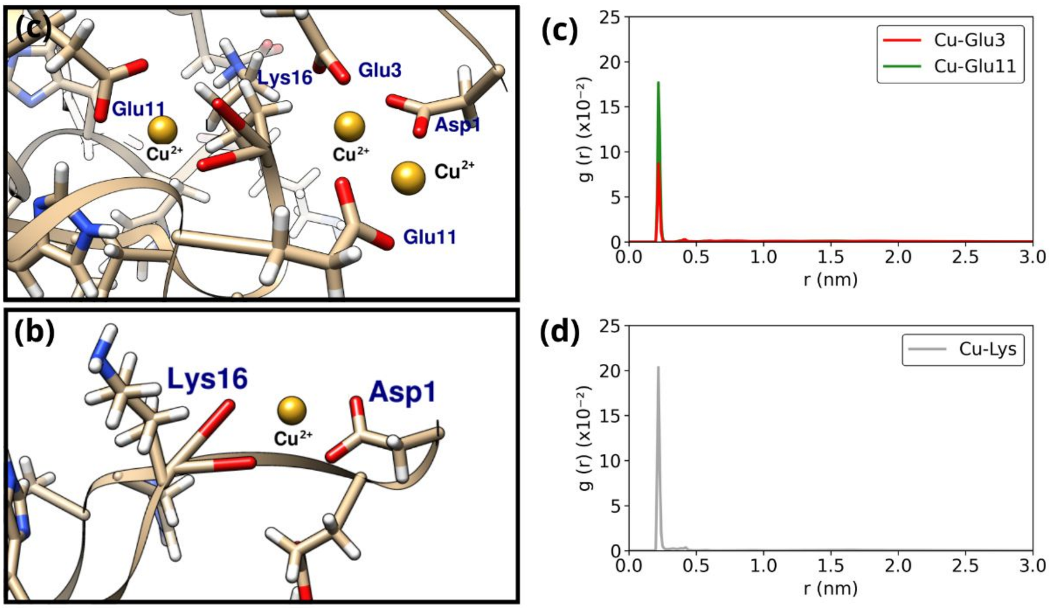

Figure S4. Representative structures obtained from simulations of the system containing 10 Aβ_{1-16} fragments and 40 Cu^{2+} ions, highlighting the main interaction modes observed between Cu^{2+} and fragments residues. (a) Interactions involving Asp1, Glu3, Glu11, and the terminal carboxylate of Lys16; (b) interactions involving Asp1 and the terminal carboxylate of Lys16. The Cu^{2+} ion is represented as a golden sphere. (c-d) Radial distribution functions (RDFs) calculated from the final 50 ns of the simulation: (c) the interaction of Cu^{2+} with Glutamate (Glu) at positions 11 (green) and 3 (red); (d) the interaction of Cu^{2+} with the terminal carboxylate of Lysine (Lys) at position 16 (gray). The g(r) values in panel (c) and (d) were multiplied by 10^{-2} for visualization purposes.

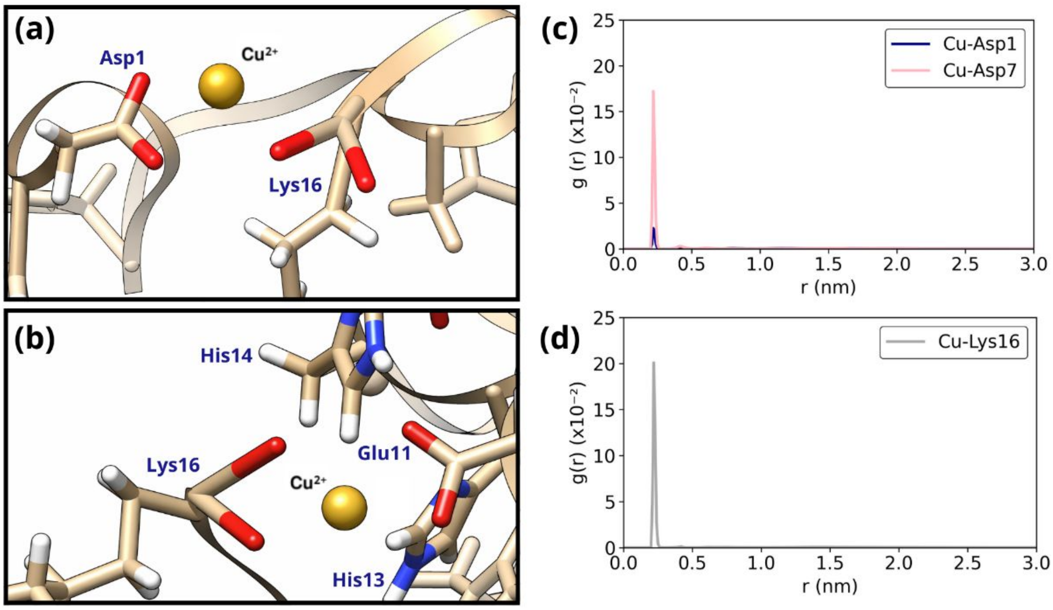

Figure S5. Representative structures obtained from simulations of the system containing 20 $A\beta_{1-16}$ fragments and 20 Cu^{2+} ions, highlighting the main interaction modes observed between Cu^{2+} and fragments residues. (a) Interactions involving Asp1 and the terminal carboxylate of Lys16; (b) interactions involving Glu11, His 13, His14 and the terminal carboxylate of Lys16. The Cu^{2+} ion is represented as a golden sphere. (c-d) Radial distribution functions (RDFs) calculated from the final 50 ns of the simulation: (c) the interaction of Cu^{2+} with Aspartate (Asp) at positions 7 (pink) and 1 (dark blue); (d) the interaction of Cu^{2+} with the terminal carboxylate of Lysine (Lys) at position 16 (gray). The g(r) values in panel (c) and (d) were multiplied by 10^{-2} for visualization purposes.

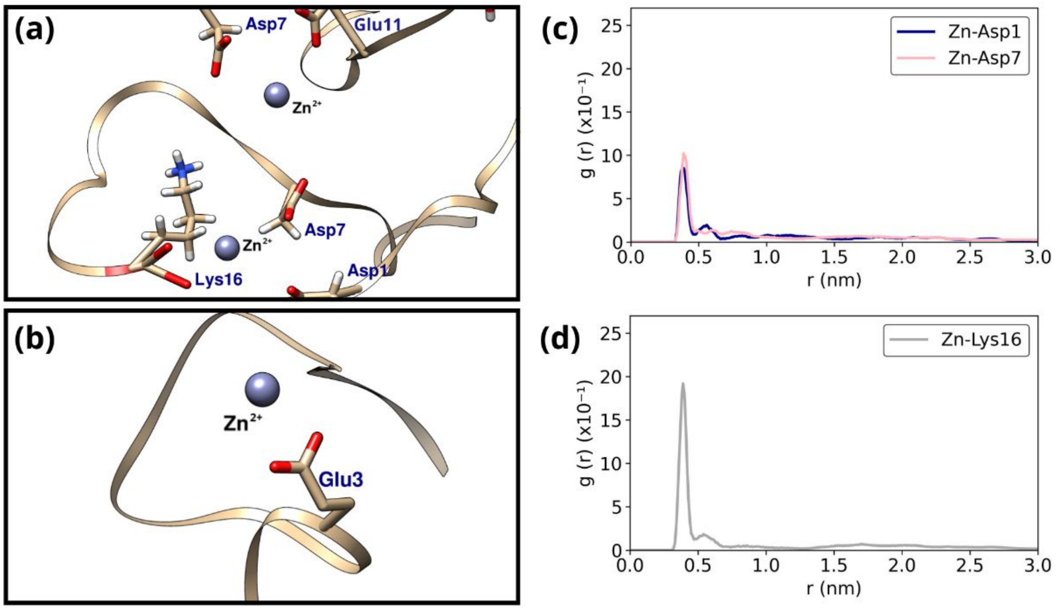

Figure S6. Representative structures obtained from simulations of the system containing 10 $A\beta_{1-16}$ fragments and 10 Zn^{2+} ions, highlighting the main interaction modes observed between Zn^{2+} and fragments residues. (a) Interactions involving Asp1, Asp7, Glu11 and the terminal carboxylate of Lys16; (b) exclusive interaction with Glu3. The Zn^{2+} ion is shown as a blue sphere. (c–d) Radial distribution functions (RDFs) calculated from the final 50 ns of the simulation: (c) the interaction of Zn^{2+} with Aspartate (Asp) at positions 7 (pink) and 1 (dark blue); (d) the interaction of Zn^{2+} with the terminal carboxylate of Lysine (Lys) at position 16 (gray). The g(r) values in panel (c) and (d) were multiplied by 10^{-1} for visualization purposes.

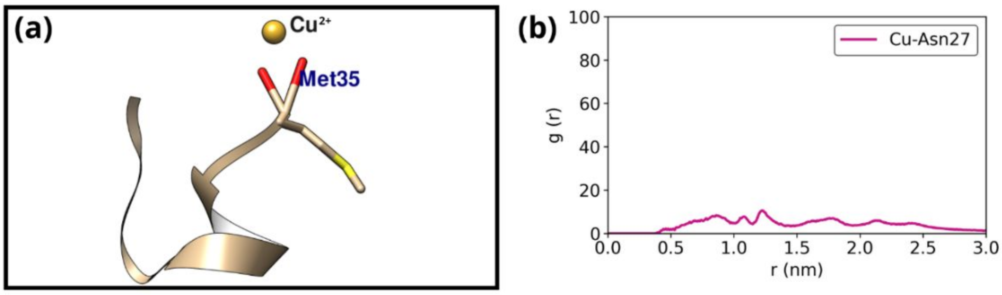

Figure S7. Representative structures obtained from the simulation of the system with 10 fragments and 10 Cu^{2+} ions, showing the main interaction modes observed between the Cu^{2+} ion and the residues of $A\beta_{25\text{-}35}$. (a) Representative structure highlighting the interaction of the Cu^{2+} ion with the terminal carboxylate group of Met35; (b) Interaction of Cu^{2+} with Asparagine (Asn) at position 27 (hot pink). Radial distribution functions (RDFs) calculated from the final 50 ns of the simulation

The use of different force fields may introduce systematic artifacts that can affect the balance between peptide–peptide and peptide–ion interactions, as well as secondary structure propensities and aggregation kinetics. In particular, differences in backbone dihedral potentials and ion parameterization schemes may lead to variations in the stability of transient conformations and in the shape of the associated free energy landscapes. Therefore, direct quantitative comparisons between systems simulated with distinct force fields should be interpreted with caution. In this work, our analysis is primarily focused on qualitative trends rather than absolute energetic differences. Accordingly, the observed differential behavior between Cu^{2+} and Zn^{2+} is discussed within this methodological limitation. To further assess the robustness of our interpretations, we also performed additional simulations of Cu^{2+} systems using the AMBER force field. As shown in Figures S8, S15, and S16, and Movie S7, the behavior obtained with AMBER is consistent with the trends observed for Zn^{2+}, while still exhibiting a higher occurrence of rare states for Cu^{2+}, in agreement with the results obtained using OPLS-AA/M. These observations support the qualitative nature of our conclusions, while reinforcing the need for caution in their quantitative interpretation.

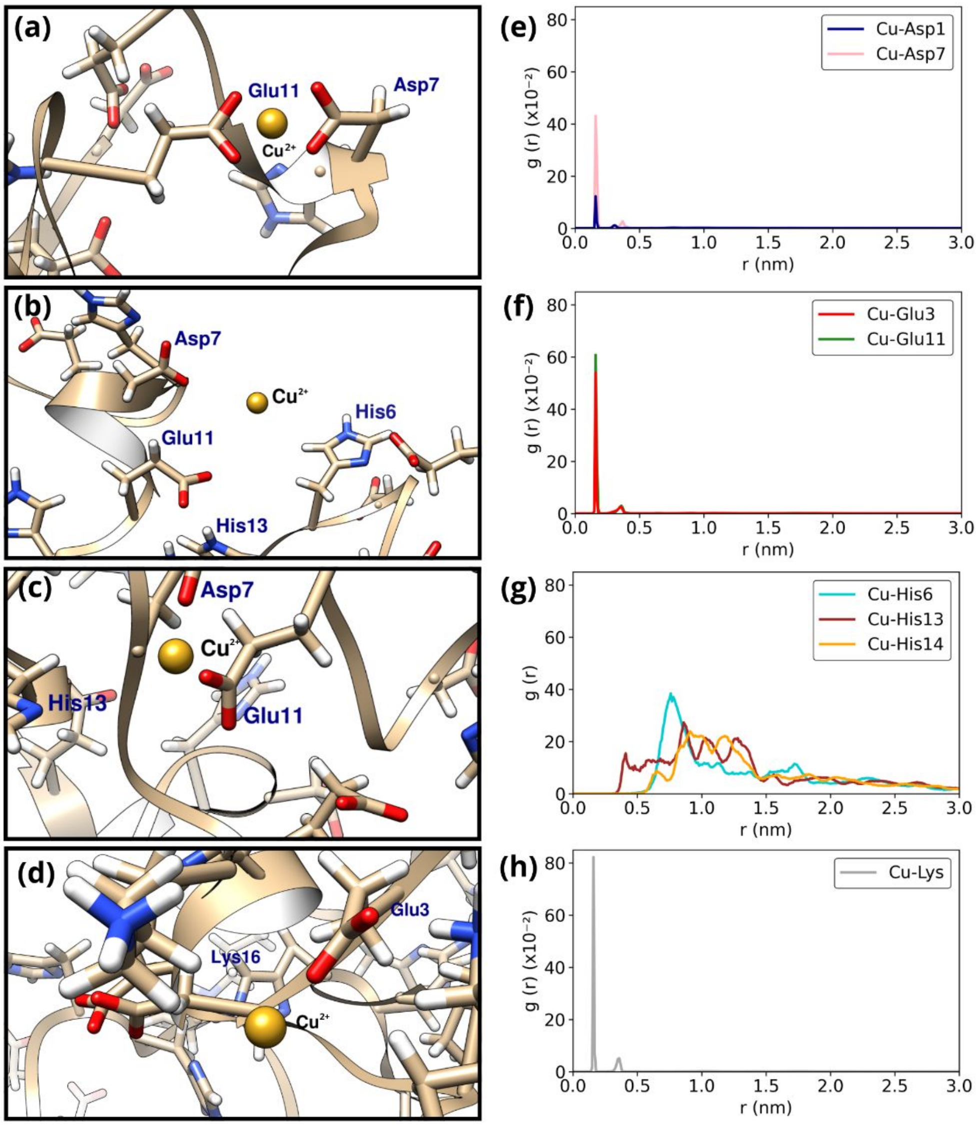

Figure S8. Representative structures obtained from simulations of the system containing 10 $A\beta_{1-16}$ fragments and 10 Cu^{2+} ions, highlighting the main interaction modes observed between Cu^{2+} and fragments residues, using the AMBER force field. (a) Interactions involving Asp7 and Glu11; (b) interactions involving Asp7, Glu11, His6 and His13; (c) interactions involving Asp7, Glu11 and His13; (d) interactions involving Glu3 and the terminal carboxylate of Lys16. The Cu^{2+} ion is represented as a golden sphere. (e-h) Radial distribution functions (RDFs) calculated from the final 50 ns of the simulation: (e) the interaction of Cu^{2+} with Aspartate (Asp) at positions 7 (pink) and 1 (dark blue); (f) the interaction of Cu^{2+} with Glutamate (Glu) at positions 11 (green) and 3 (red); (g) interactions of Cu^{2+} with histidine residues His6 (light blue), His13 (dark red), and His14 (orange); (h) the interaction of Cu^{2+} with the terminal carboxylate of Lysine (Lys) at position 16 (gray). The g(r) values in panel (e), (f) and (h) were multiplied by 10^{-2} for visualization purposes.

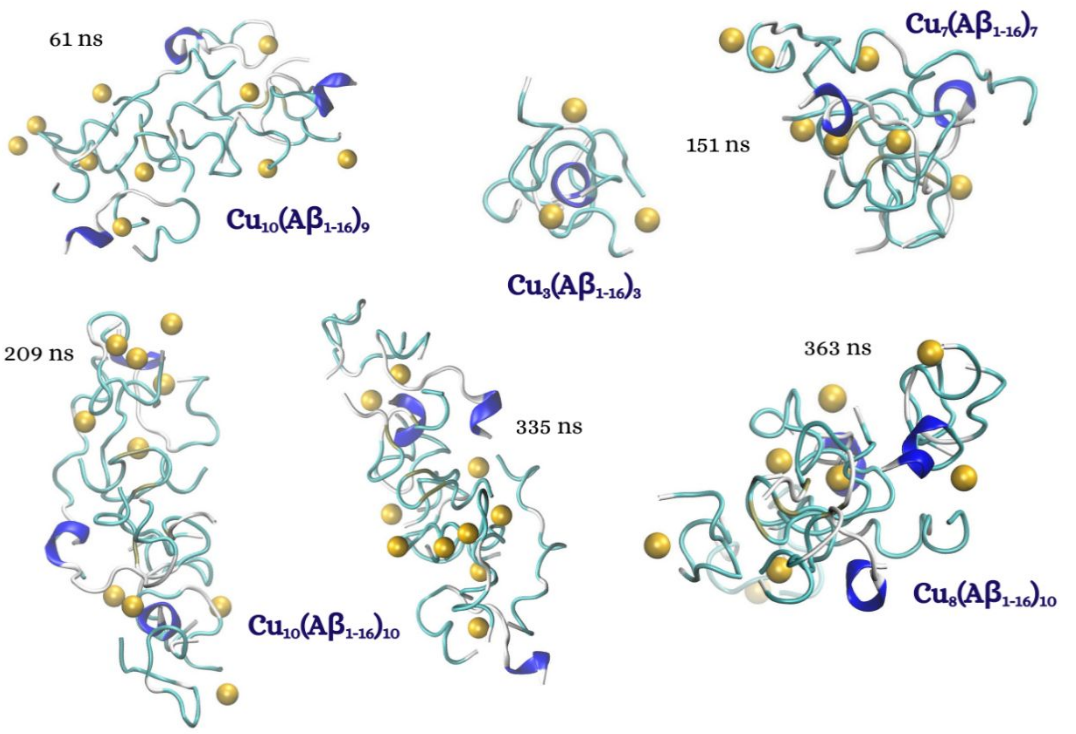

Figure S9. Snapshots from the molecular dynamics trajectory of 10 $A\beta_{1\text{-}16}$ fragments in the presence of 10 Cu^{2+} ions (gold spheres), showing the formation of proto-oligomers species with varying aggregation numbers at different simulation times.

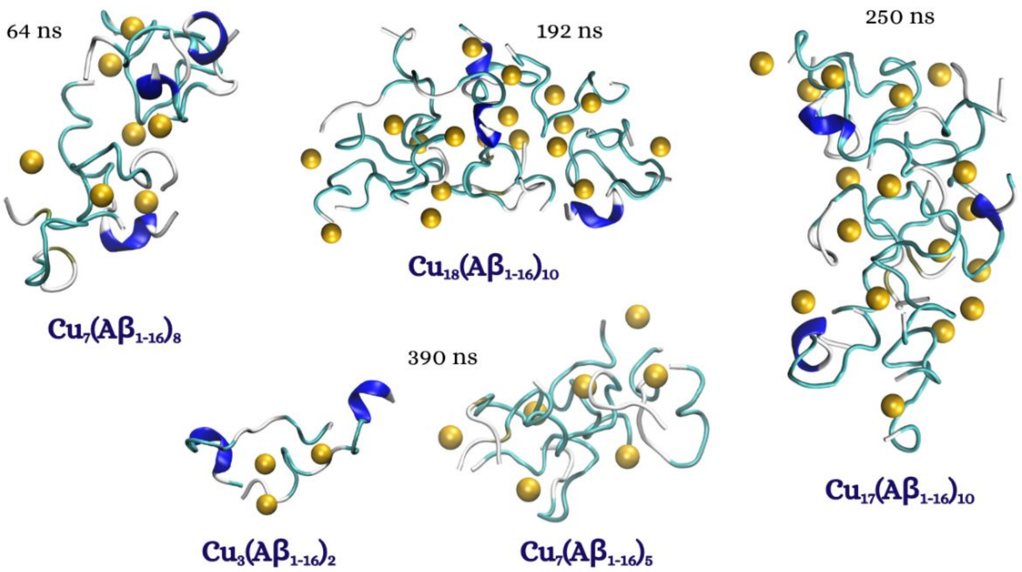

Figure S10: Snapshots from the molecular dynamics trajectory of 10 $A\beta_{1\text{-}16}$ fragments in the presence of 20 Cu^{2+} ions (gold spheres), showing the formation of proto-oligomers species with varying aggregation numbers at different simulation times.

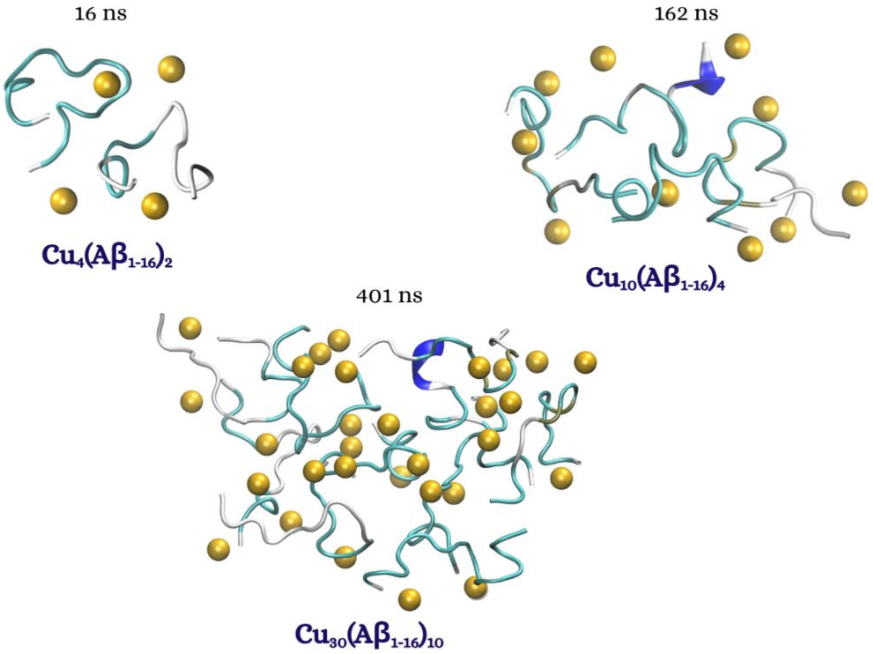

Figure S11: Snapshots from the molecular dynamics trajectory of 10 $A\beta_{1-16}$ fragments in the presence of 40 Cu^{2+} ions (gold spheres), showing the formation of proto-oligomers species with varying aggregation numbers at different simulation times.

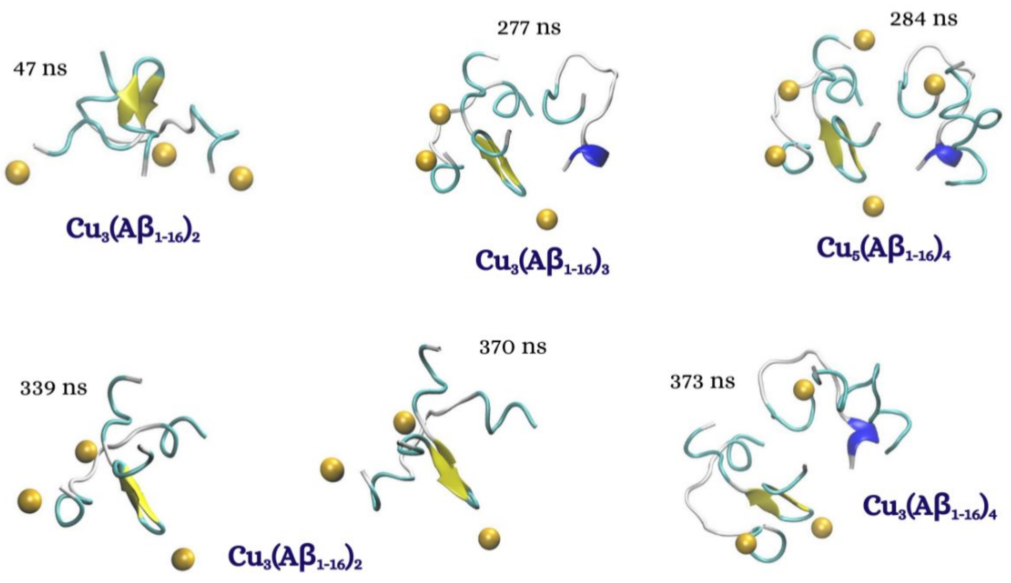

Figure S12. Snapshots from the molecular dynamics trajectory of 20 $A\beta_{1-16}$ fragments in the presence of 20 Cu^{2+} ions (gold spheres), showing the formation of proto-oligomers species with varying aggregation numbers at different simulation times.

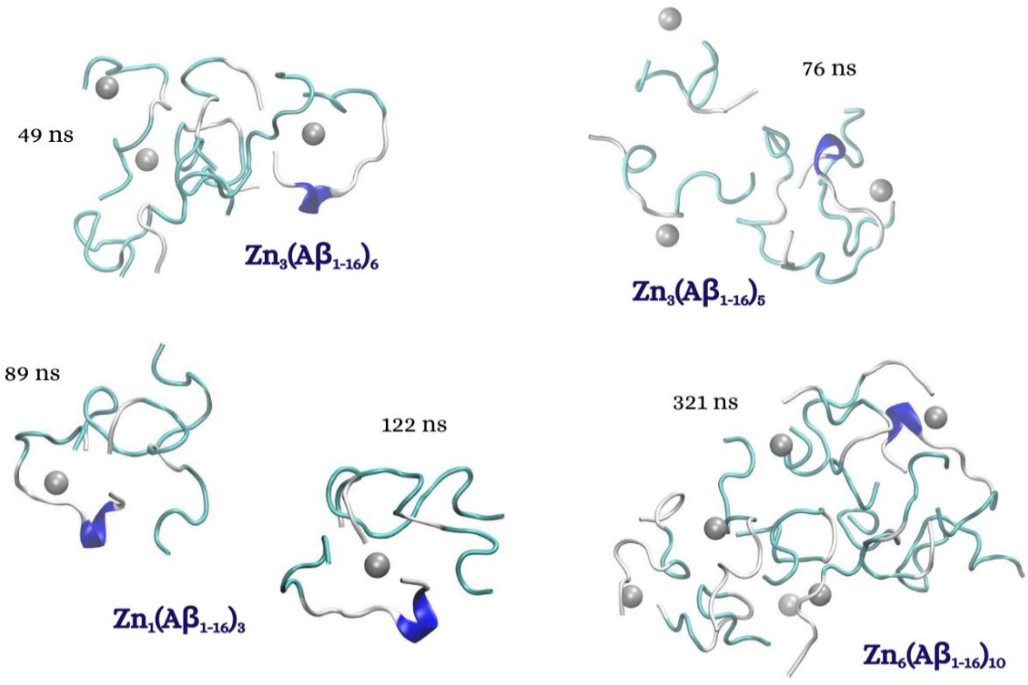

Figure S13. Snapshots from the molecular dynamics trajectory of 10 $A\beta_{1\text{-}16}$ fragments in the presence of 10 Zn^{2+} ions (blue sphere), showing the formation of proto-oligomers species with varying aggregation numbers at different simulation times.

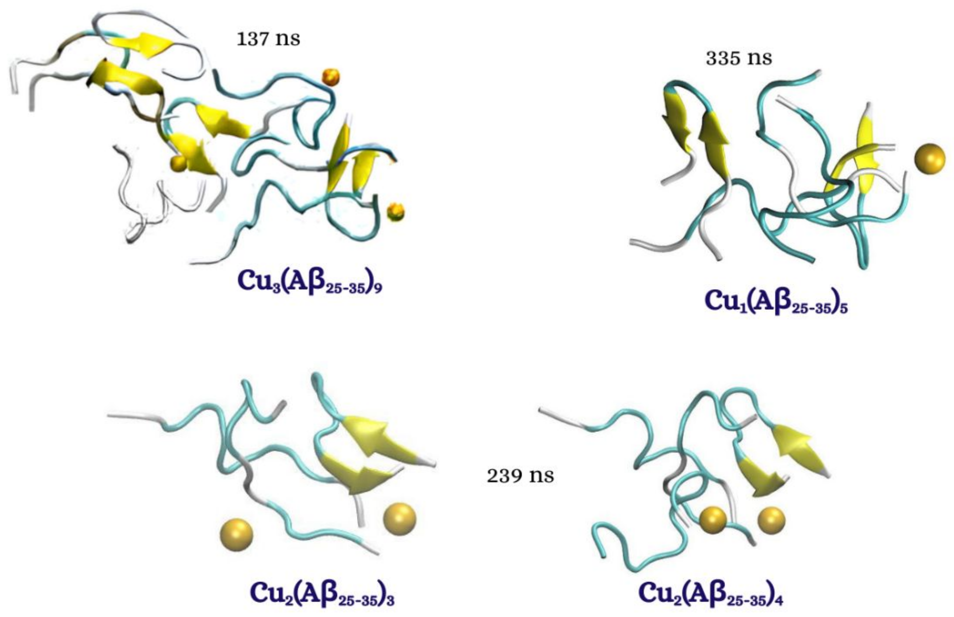

Figure S14. Snapshots from the molecular dynamics trajectory of 10 $A\beta_{25-35}$ fragments in the presence of 10 Cu^{2+} ions (gold spheres), showing the formation of proto-oligomers species with varying aggregation numbers at different simulation times.

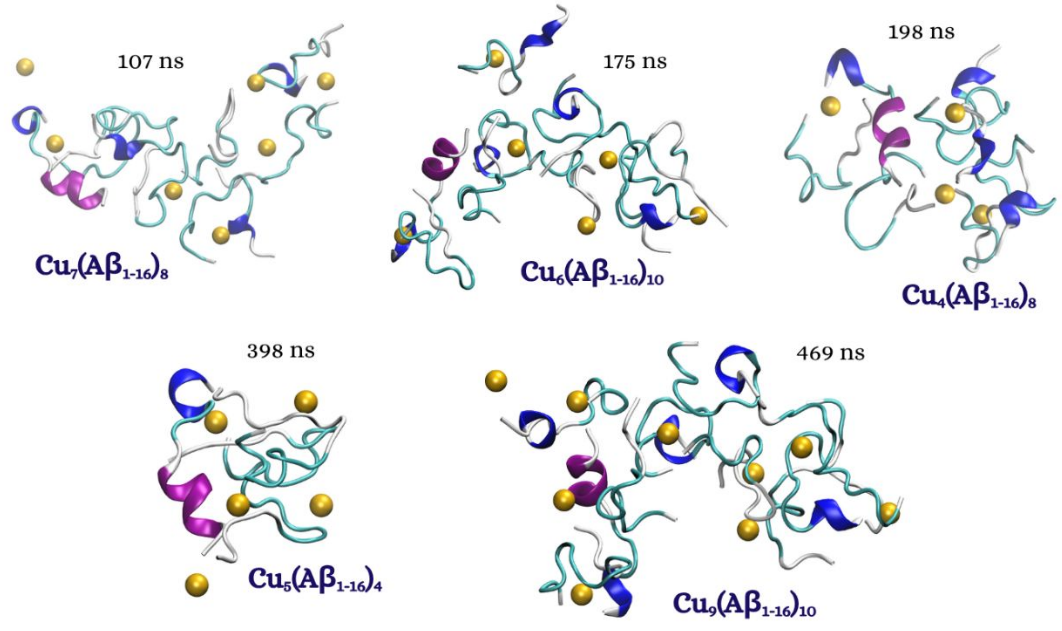

Figure S15: Snapshots from the molecular dynamics trajectory of 10 A$\beta_{1\text{-}16}$ fragments in the presence of 10 Cu^{2+} ions (gold spheres), showing the formation of proto-oligomer species with varying aggregation numbers at different simulation times, obtained using the AMBER force field for comparison purposes.

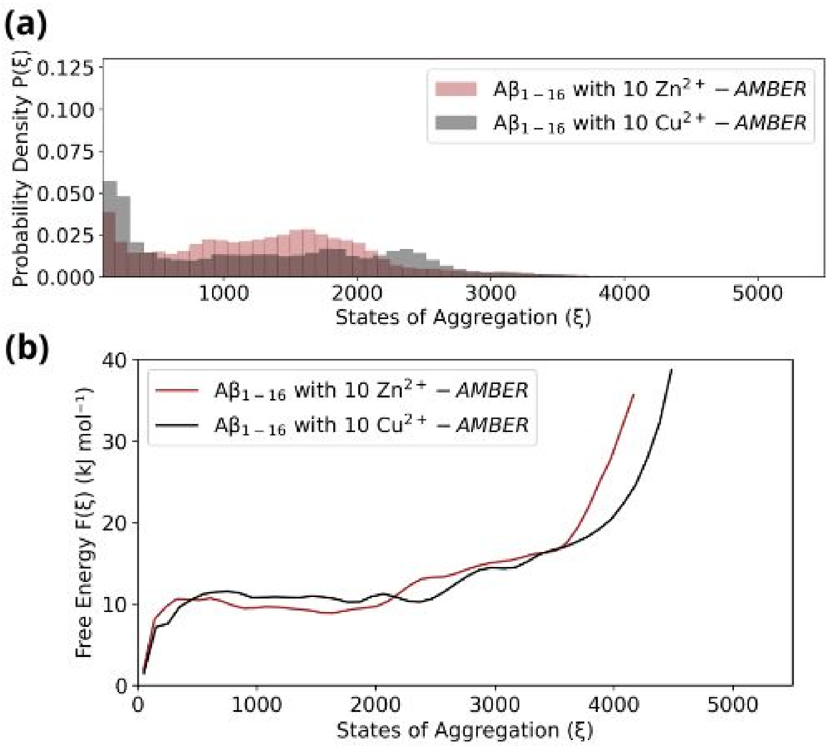

Figure S16: (a) Probability distribution graph for the collective variable aggregation. (b) Energy required to access the aggregation states. Comparison between aggregation simulations of the $A\beta_{1\text{-}16}$ fragment in aqueous solution with Cu^{2+} and Zn^{2+} ions using the AMBER force field.

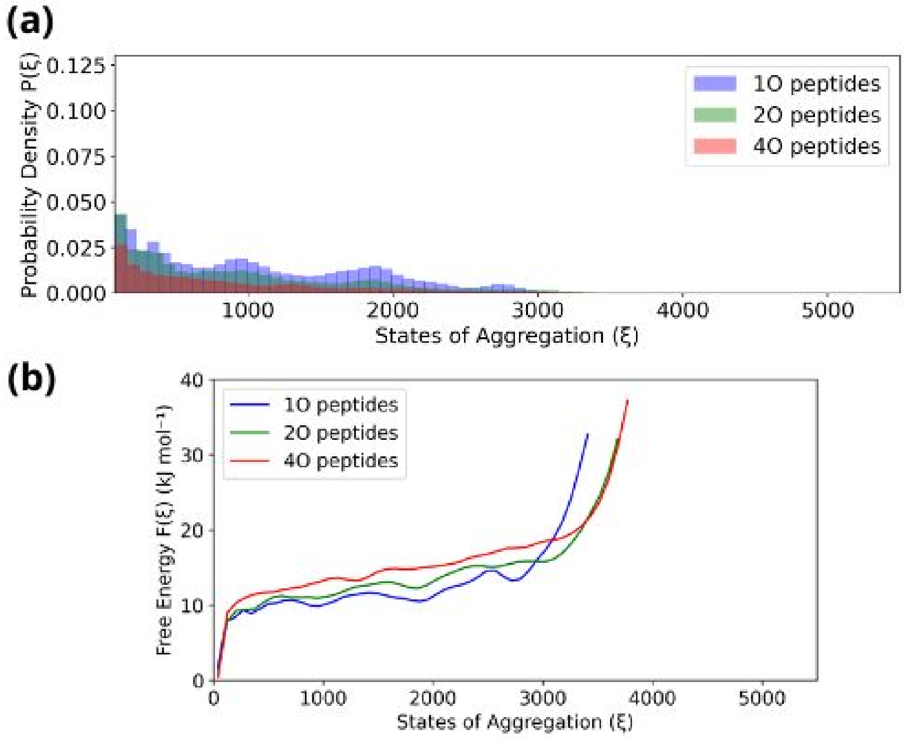

Figure S17: (a) Probability distribution graph for the collective variable aggregation. (b) Energy required to access the aggregation states. Comparison between simulations of aggregation of the A$\beta_{25\text{-}35}$ fragment in aqueous solution with 10, 20 and 40 without ions.

Summary of Movie captions

Movie S1: Molecular dynamics trajectory of the system containing 10 $A\beta_{1-16}$ fragments in the presence of 10 Cu^{2+} ions, illustrating the structural evolution and aggregation events throughout the simulation. The Cu^{2+} ion is represented as a golden sphere.

Movie S2: Molecular dynamics trajectory of the system containing 10 $A\beta_{1-16}$ fragments in the presence of 20 Cu^{2+} ions, illustrating the structural evolution and aggregation events throughout the simulation. The Cu^{2+} ion is represented as a golden sphere.

Movie S3: Molecular dynamics trajectory of the system containing 10 $A\beta_{1-16}$ fragments in the presence of 40 Cu^{2+} ions, illustrating the structural evolution and aggregation events throughout the simulation. The Cu^{2+} ion is represented as a golden sphere.

Movie S4: Molecular dynamics trajectory of the system containing 20 $A\beta_{1-16}$ fragments in the presence of 20 Cu^{2+} ions, illustrating the structural evolution and aggregation events throughout the simulation. The Cu^{2+} ion is represented as a golden sphere.

Movie S5: Molecular dynamics trajectory of the system containing 10 $A\beta_{1-16}$ fragments in the presence of 10 Zn^{2+} ions, illustrating the structural evolution and aggregation events throughout the simulation. The Zn^{2+} ion is shown as a blue sphere.

Movie S6: Molecular dynamics trajectory of the system containing 10 $A\beta_{25-35}$ fragments in the presence of 10 Cu^{2+} ions, illustrating the structural evolution and aggregation events throughout the simulation. The Cu^{2+} ion is represented as a golden sphere.

Movie S7: Molecular dynamics trajectory of the system containing 10 $A\beta_{1-16}$ fragments in the presence of 10 Cu^{2+} ions, illustrating the structural evolution and aggregation events

throughout the simulation, using the AMBER force field for comparison purposes. The Cu^{2+} ion is represented as a golden sphere.
